# Robust load-frequency control of islanded urban microgrid using 1PD-3DOF-PID controller including mobile EV energy storage

**DOI:** 10.1038/s41598-024-64794-y

**Published:** 2024-06-17

**Authors:** Iraj Faraji Davoudkhani, Peyman Zare, Almoataz Y. Abdelaziz, Mohit Bajaj, Milkias Berhanu Tuka

**Affiliations:** 1 Department of Electrical Engineering, University of Mohaghegh Ardabili, Ardabil, Iran; 2 Faculty of Engineering, Ain Shams University, Cairo, 11517 Egypt; 3 Faculty of Engineering and Technology, Future University in Egypt, Cairo, 11835 Egypt; 4 Department of Electrical Engineering, Graphic Era (Deemed to be University), Dehradun, 248002 India; 5 Hourani Center for Applied Scientific Research, Al-Ahliyya Amman University, Amman, Jordan; 6 Graphic Era Hill University, Dehradun, 248002 India; 7 Department of Electrical and Computer Engineering, College of Engineering, Addis Ababa Science and Technology University, Addis Ababa, Ethiopia

**Keywords:** Islanded urban microgrid, Mobile electric vehicle energy storage, Energy storage systems, 1PD-3DOF-PID cascade controller, Coati optimization algorithm, Load frequency control, Energy science and technology, Mathematics and computing, Engineering, Electrical and electronic engineering

## Abstract

Electricity generation in Islanded Urban Microgrids (IUMG) now relies heavily on a diverse range of Renewable Energy Sources (RES). However, the dependable utilization of these sources hinges upon efficient Electrical Energy Storage Systems (EESs). As the intermittent nature of RES output and the low inertia of IUMGs often lead to significant frequency fluctuations, the role of EESs becomes pivotal. While these storage systems effectively mitigate frequency deviations, their high costs and elevated power density requirements necessitate alternative strategies to balance power supply and demand. In recent years, substantial attention has turned towards harnessing Electric Vehicle (EV) batteries as Mobile EV Energy Storage (MEVES) units to counteract frequency variations in IUMGs. Integrating MEVES into the IUMG infrastructure introduces complexity and demands a robust control mechanism for optimal operation. Therefore, this paper introduces a robust, high-order degree of freedom cascade controller known as the 1PD-3DOF-PID (1 + Proportional + Derivative—Three Degrees Of Freedom Proportional-Integral-Derivative) controller for Load Frequency Control (LFC) in IUMGs integrated with MEVES. The controller’s parameters are meticulously optimized using the Coati Optimization Algorithm (COA) which mimics coati behavior in nature, marking its debut in LFC of IUMG applications. Comparative evaluations against classical controllers and algorithms, such as 3DOF-PID, PID, Reptile Search Algorithm, and White Shark Optimizer, are conducted under diverse IUMG operating scenarios. The testbed comprises various renewable energy sources, including wind turbines, photovoltaics, Diesel Engine Generators (DEGs), Fuel Cells (FCs), and both Mobile and Fixed energy storage units. Managing power balance in this entirely renewable environment presents a formidable challenge, prompting an examination of the influence of MEVES, DEG, and FC as controllable units to mitigate active power imbalances. Metaheuristic algorithms in MATLAB-SIMULINK platforms are employed to identify the controller’s gains across all case studies, ensuring the maintenance of IUMG system frequency within predefined limits. Simulation results convincingly establish the superiority of the proposed controller over other counterparts. Furthermore, the controller’s robustness is rigorously tested under ± 25% variations in specific IUMG parameters, affirming its resilience. Statistical analyses reinforce the robust performance of the COA-based 1PD-3DOF-PID control method. This work highlights the potential of the COA Technique-optimized 1PD-3DOF-PID controller for IUMG control, marking its debut application in the LFC domain for IUMGs. This comprehensive study contributes valuable insights into enhancing the reliability and stability of Islanded Urban Microgrids while integrating Mobile EV Energy Storage, marking a significant advancement in the field of Load-Frequency Control.

## Introduction

### Background and challenges

Cities face power shortages due to fossil fuel depletion, energy demands, and environmental concerns, requiring global diversification of electricity generation methods^1^. Due to increasing global temperatures and climate change consequences, there’s a growing push to shift to Renewable Energy Sources (RESs) and replace internal combustion engines with Electric Vehicles (EVs)^2^. The influence rise of RES, EVs, and controllable loads has led to Islanded Urban Microgrids (IUMGs). These systems operate independently but integrating RES presents sustainability challenges. Thus, effective integration into IUMGs demands comprehensive planning^1–3^. The IUMG faces vulnerability due to low inertia and intermittent RES power generation. This susceptibility can disrupt system frequency, potentially causing cascading failures and system collapse^3^. IUMGs utilize various energy storage technologies to address power generation-consumption disparities and frequency fluctuations. Energy Storage Systems (ESSs) are crucial for preventing power imbalances and providing swift response to load variations. Battery Energy Storage Systems (BESS) and Flywheel Energy Storage Systems (FESS) are particularly effective in this regard^4,5^. The feasibility of this capability is attributed to the technology’s high energy density and bidirectional power regulation during both charging and discharging. Mobile EV Energy Storage (MEVES) functions as a mobile ESS, providing ancillary services to IUMGs^6,7^. Hence, the role played by MEVES in facilitating frequency control within IUMGs is steadily gaining prominence and becoming increasingly conspicuous^8–14^. Furthermore, MEVES gains popularity due to cost-effective charging, reduced fossil fuel reliance, and minimal emission^8,9^. Nonetheless, the steadily growing fleet of interconnected MEVES units, each equipped with storage batteries, assumes a substantial role in IUMGs^10^. The importance of this functionality will grow with the rapid spread of MEVES in IUMGs. MEVES batteries have the potential to reduce daily load profiles and mitigate peak loads^8,10,11^. MEVES’s involvement in IUMGs primarily aims to reduce frequency deviations through Load Frequency Control (LFC), maintaining system frequency within defined limits by managing power supply–demand disparities. MEVES, with its charging and discharging capabilities, enhances frequency control in IUMGs, necessitating efficient and robust frequency controller design for stable operation amid renewable energy integration and MEVES adoption^8,13,15^.

### Literature survey

Global researchers aim to improve Microdrid (MG) frequency control for stable frequency profiles post-load disturbances. LFC enhancements can be based on system performance, controller structure, and optimization methods^16,17^. Some studies use advanced algorithms, explore novel controller regulations, and introduce a hybrid controller with a novel optimizer. This paper’s strategy employs a new optimization algorithm combined with a hybrid controller, a valuable endeavor in the quest for superior LFC mechanisms.

Numerous control strategies for LFC in conventional MGs are crucial for operation and control, including integer-order, fractional-order, fuzzy-logic-based, data-driven, and model-predictive-based LFCs^18,19^. To LFC, integer-order frequency controllers are commonly used. These controllers are constructed using various configurations, including combinations of Proportional (P), Integral (I), and Derivative (D) branches, with or without filters (F)^20^.

Literature explores various methodologies for designing integer-order frequency controllers, using control theory, analytical techniques, and optimization algorithms. PID controller derivatives are preferred for simplicity, reliability, and cost-effectiveness^21^. Authors in^22,23^ implemented a conventional PI controller and in^24^ conventional PID for the LFC analysis. In^25^, A LFC method based on standard PI controllers is suggested for quick setup, but stability issues arise due to communication system delays. In addition, the PI-based LFC technique described in^26^ was developed with the help of the harris hawks optimizer algorithm. In^27^ suggests using a particle swarm optimization algorithm to build a virtual inertia PI controller for a single-area Egyptian power system. An integer order based dual stage LFC approach is proposed in^28^, and the Binary butterfly optimization is used in developing of the controller for this technique.

Integer order LFCs manage RESs intermittency but cannot completely reduce frequency variations in power networks like conventional PI/PID controllers^29^.

Fractional-order LFC methods are gaining prominence in addressing frequency control challenges, including integration with traditional PI/PID controllers for optimization in challenging scenarios^30–32^. In addition, higher Degrees of Freedom (DoF) control techniques for LFC issues, such as Two-Degree of Freedom (2DoF) and Three-Degree of Freedom (3DoF), have been recently published in the research literature^33,34^. Higher DoF controllers enhance responsiveness but complicate design with sensors, delays, and complexities; research suggests modified and hybrid structures^33,34^.

The authors assess single DoF PID and FOPID performance compared to LFCs 2DOF-PID, 3DOF-PID, 2DOF-FOPID, and 3DOF-FOPID. They recommend cascaded controllers for reducing LFC response in MGs^35,36^. Other controllers, such as robust controllers^37,38^, have also been successfully develope for the LFC analysis of MGs to these.

In addition, advanced LFC methods that are based on model predictive control have been developed and documented in the relevant academic literature^39^. Theories of model predictive control, both centralized and decentralized, have been proposed as solutions to LFC problems^40^. Other intelligent LFC systems have been proposed, such as robust LFC systems^41^, internal model LFCs^42^, sliding mode LFCs^43^, adaptive LFCs^44^, intelligent LFC systems^45^, linear-matrix-inequalities LFCs^46^.

Researchers use metaheuristic optimization algorithms to enhance controller responsiveness in LFCs, focusing on precise parameter adjustments for improved system performance. On the other hand, mobile ESS in MGs introduce complexities to LFC challenges; ensuing discussion explores LFC studies considering these devices.

A fuzzy-based LFC method for distributed megawatt-class PV systems considering EVs was presented in^47^. A multiple MPC strategy was given for MG frequency stability in^48^, considering A plug-in hybrid EVs bidirectional charging and discharging and state-of-charge regulation. The autonomous distributed vehicle-to-grid control LFC method was introduced in^49^. The plan suggested in^49^ did not ensure that the vehicle-to-grid and LFC controllers would have a synchronized control effect. Therefore, in^50^, a reliable H_2_ /H_∞_ LFC controller was presented to solve this issue. A robust and adaptable LFC system for use by MGs in the presence of EVs was given in reference^51^. The paper^52^ discusses a reliable frequency control technique for an islanded MG that uses the H_∞_ and μ-synthesis methods. The process suggested in^52^ is complicated to implement and will only function as desired if the precise mathematical model of the plant is available^29,53^.

Recent research on hybrid approaches, especially high DoF cascade controllers, in LFC problem-solving shows potential for enhanced performance. Optimization methods are crucial for selecting optimal gain values for MG-based LFCs. Table 1 compares meta-heuristic optimization techniques and controllers, highlighting their contributions.

**Table 1:** Summarized literature review and the paper contributions**.**

Refs.	Components system							Control method		Methodology Optimization		Contribution
	RES		DER		ESS							
	Solar	Wind	DEG	FC	Non-mobile		mobile	Mian	Comparison	Mian	Comparison	
					BESS	FESS	EVs					
^10^	✓	✓	✓	✓	✓	✓	✓	PI-PD	I,PI,PID	SSO	PSO,GWO	The proposed PI-PD cascade controller excels with low performance index values and robustness to parameter variations
^54^	✓	✓	✓	×	✓	✓	✓	AIMPC	AFMPC ,PID	DSCA	–	A novel AIMPC scheme enhances MG LFC by optimizing tuning parameters and controlling EV batteries
^55^	✓	✓	✓	✓	✓	✓	✓	FPD-TID	IOPID,FPID,TID,CC-TID	CCSA	–	This paper proposes a hybrid FPD-TID controller for SMG LFC with better FDR and robustness to parameter uncertainties
^56^	✓	✓	✓	✓	✓	✓	✓	FNSMC	PID, FPI ,MPC	MBHA	–	Paper presents optimal nonlinear LFC controller with low computational burden
^57^	×	×	×	×	×	×	✓	I^λ^D^μ^F	PIDF	FPA	–	Examines fractional-order LFC controller in integrated EV fleets, demonstrating superior simulation performance
^51^	✓	✓	✓	✓	✓	✓	✓	GT2FPI	OFPI,IT2FPI	MHSA	–	A new time-varying controller based on General Type II Fuzzy Logic for LFC in an isolated micro-grid with V2G technique
^29^	×	✓	✓	×	×	×	✓	T2FPI	PID,FPID,IT2FPI	MHSA	–	The OGT2FPI is a novel intelligent adaptive PI controller designed to reduce power fluctuations and load disturbances in islanded MicroGrids
^58^	×	×	×	×	×	×	✓	PI-PD	PI, PID	hSFS-PS	–	hSFS-PS optimized cascade PI-PD controller enhances multi-unit PEV system response
^35^	×	×	×	×	×	×	✓	PI-PD	PI-PD	MGWO	GSA,DE, PSO, GWO	This paper introduces a novel fully-distributed control strategy for effective coordination and fast frequency regulation in islanded microgrids
^59^	×	✓	✓	×	×	×	×	DCS-ILADRC	CCS-ILADRC	GA- PSO	–	Novel distributed control strategy for fast frequency regulation and efficient distributed energy coordination in islanded microgrids
This paper	✓	✓	✓	✓	✓	✓	✓	1PD-3DOF-PID	3DOF-PID,PID	COA	RSA,WSO	The COA Technique-optimized 1PD-3DOF-PID controller is a novel optimization method based on natural coati behavior

### Researchs gap

The growing dependence on RES in IUMGs presents a significant challenge: maintaining stable frequency due to the inherent variability of RES power generation and the low inertia of these MGs. While FC strategies have been extensively researched for traditional power grids and MGs, a review of the recent literature reveals specific gaps in the context of IUMGs:

1. **Limited exploration of hybrid variable controllers:** Existing LFC approaches for IUMGs primarily focus on classical controllers or integer-order controllers. These controllers may not be fully equipped to handle the complex dynamics of IUMGs with significant RES penetration. The potential of more sophisticated control strategies, such as hybrid variable controllers that combine different control structures, remains largely unexplored in IUMG LFC. These hybrid controllers have the potential to offer improved performance by leveraging the strengths of different control paradigms and adapting to the varying operating conditions in IUMGs.
2. **Need for IUMG-specific optimization algorithms:** The design of frequency control systems in various power systems and MGs often relies on heuristic and meta-heuristic optimization methods for optimal controller parameter selection. However, these traditional optimization algorithms may not be tailored to the unique dynamics of IUMGs. While some studies have investigated meta-heuristic optimization algorithms for LFC in traditional microgrids, their application in IUMG LFC is limited. Integrating optimization algorithms specifically designed for the dynamic characteristics of IUMGs can lead to more robust and adaptable LFC strategies, ensuring superior frequency regulation performance.
3. **Underexplored potential of MEVES:** The integration of EV batteries as MEVES units for frequency regulation in IUMGs is a novel concept with significant potential. The MEVES offers a promising approach to address the challenges associated with RES variability and low inertia in IUMGs. However, existing research has not fully explored the optimal control strategies and optimization techniques necessary to effectively leverage MEVES for LFC in IUMGs. Developing robust control algorithms and optimization methods tailored to MEVES integration is crucial to unlock the full potential of this technology for enhancing frequency stability in IUMGs.

### Research motivation

This research is motivated by the need for enhanced LFC strategies in IUMGs. Existing methods may not adequately address the complex dynamics of these microgrids, particularly those with high penetration of RES. Here, the inherent variability of RES power generation poses significant challenges for maintaining frequency stability. This paper proposes a novel and robust cascade hybrid controller specifically designed for precise frequency regulation in IUMGs. The controller is strategically placed within the secondary control level, providing a powerful tool for mitigating frequency deviations. Additionally, a unique meta-heuristic optimization method is implemented to optimize the controller gains, ensuring optimal performance under varying operating conditions.

### Contributions

A review of the existing literature reveals that numerous studies have explored LFC approaches in MGs powered by RES. Notably, some of them have often employed mobile ESS or EV models for their analyses within power systems and MGs^10,10,29,35,51,54–60^.

After thorough investigation, it becomes evident that the inclusion of EVs positively impacts LFC in MGs. Encouraged by this insight, the authors present this current LFC study, incorporating MEVES systems. In the next phase of innovation for constructing the LFC loop within an IUMG, the exploration of higher DoF-based control approaches holds significant promise. Among the available controllers, higher DoF-based control strategies, particularly cascade controllers, excel in mitigating frequency deviations while simplifying system complexity. Recent advancements in metaheuristic-based design methods and cascade control-based DoF for LFC have showcased superior performance when compared to classical design methods and integer-order controllers.

These attributes of higher DoF-based control approaches serve as the inspiration behind the integration into a proposed enhanced cascade controller, referred to as the higher DoF Cascade Controller. This controller combines the advantages of the 3DOF (Three Degree Of Freedom (3DOF) Proportional (P)-Integral (I)-Derivative (D) (PID)) and 1PD (1 + Proportional (P) + Derivative(D)) controller, offering improved frequency regulation capabilities for IUMGs. The proposed controller is distinguished by its unique approach, drawing inspiration from the amalgamation of the best attributes of 3DOF and 1PD controllers.

Achieving precise frequency control relies on controllers designed to their fullest potential. To enhance this controller’s performance, a novel metaheuristic optimization algorithm known as the Coati Optimization Algorithm (COA)^61^ has been developed and implemented. This algorithm serves to fine-tune the coefficients of the recommended controller, thereby optimizing its performance. The performance index in the time domain serves as a composite measure of frequency and time changes, forming the objective function in the design process. This function, which undergoes reduction during the optimization process, plays a pivotal role in the design process.

A performance comparison was carried out, pitting the proposed controller against established counterparts such as 3DOF-PID and PID, as well as algorithms like the Reptile Search Algorithm (RSA)^62^ and White Shark Optimizer (WSO)^63^. Despite these noteworthy origins, the COA optimization technique emerges as the predominant optimizer in this study, owing to its diverse advantages.

The WSO and RSA are optimization algorithms inspired by great white sharks’ sensory abilities and crocodiles’ social hunting behavior. COA, a competitive algorithm, models predatory tactics and evasion strategies in coatis, focusing on exploration and exploitation phases. COA technique improves global optimization by eliminating control parameters, reducing adjustments, and balancing exploration and exploitation, delivering outstanding performance in real-world applications. Hence, the proposed technique offers ease of implementation without the need for controlling parameters. Users only require the fitness function for optimization, distinguishing it from labor-intensive traditional tuning procedures that often underperform across various system operating conditions. In recent times, researchers have employed the COA method^64,65^ to address a range of challenging optimization problems.

This discovery has motivated the authors to propose the COA approach for LFC analysis within an IUMG. To the best of the authors’ knowledge, there are no instances of implementing the recommended approach for LFC analysis in the existing literature. This comprehensive assessment encompassed a range of operational scenarios within the IUMG to provide substantial evidence supporting the adequacy of the recommended control method.

Motivated by the filling of research gaps and the aim to contribute innovative research in alignment with recent advancements in LFC methods, particularly those grounded bdaed cascade control-based DoF theory, this paper presents several significant contributions:

1. **Unleashing the Power of Hybrid Variable Controllers**
  **Research Gap: Limited Exploration of Hybrid Variable Controllers:** Conventional LFC approaches in IUMGs frequently rely on classical or integer-order controllers. These controllers, while established, might not possess the necessary sophistication to effectively manage the intricate dynamics of IUMGs characterized by high RES penetration and inherently low inertia. This limitation can manifest as sluggish response times and an inability to adequately dampen frequency deviations.
  **Innovation:** To circumvent these shortcomings, this paper introduces a groundbreaking 1PD-3DOF-PID hybrid controller. This ingenious controller design strategically merges the strengths of two well-regarded control paradigms: 1PD and 3DOFPID. This synergistic amalgamation empowers the controller to deliver superior LFC performance by:
    **Minimizing Overshoot/Undershoot:** By meticulously regulating the controller’s response characteristics, the 1PD-3DOF-PID controller effectively curtails the magnitude of frequency excursions, preventing excessive overshoot or undershoot during load or generation imbalances.
    **Attenuating Transient Effects**: The robust design of the controller enables it to swiftly counteract sudden fluctuations in power generation or demand, thereby minimizing transient effects that can disrupt grid stability.
    **Enhancing Response Speed:** The 1PD-3DOF-PID controller boasts a faster response time compared to traditional controllers. This agility allows for more rapid interventions to stabilize frequency imbalances and maintain grid integrity.
2. **IUMG-Centric Optimization: A Tailored Approach**
  **Research Gap _ Need for IUMG-Specific Optimization Algorithms:** Current optimization algorithms employed for LFC design might not be specifically calibrated to the unique dynamical characteristics of IUMGs. This mismatch can lead to suboptimal controller performance and hinder the effectiveness of LFC strategies.
  **Innovation:** This paper proposes the COA, a novel optimization technique meticulously crafted to address the specific requirements of IUMGs. The COA algorithm excels in:
    **Extracting Optimal Controller Parameters**: The COA algorithm meticulously explores the parameter space to identify the optimal configuration for the 1PD-3DOF-PID controller. This ensures the controller functions at its peak efficiency under diverse IUMG operating conditions, maximizing its effectiveness in real-world scenarios.
    Enhanced Robustness and Adaptability: Unlike conventional optimization algorithms, the COA is specifically designed to handle the dynamic nature of IUMGs. This inherent robustness allows the COA to adapt to fluctuating system conditions and consistently deliver optimal controller parameters.
3. **Unveiling the Full Potential of MEVES Integration**
  **Research Gap _ Underexplored Potential of MEVES:** While MEVES presents a promising avenue for LFC in IUMGs, a comprehensive understanding of optimal control strategies and optimization techniques for its effective utilization remains elusive.
  **Innovation:** This paper proposes a pioneering control strategy specifically tailored for linked MEVES that leverages the capabilities of the 1PD-3DOF-PID controller. This strategic approach aims to:
    **Optimizing MEVES Utilization:** The control strategy meticulously coordinates the participation of MEVES units within the LFC framework, ensuring their efficient deployment for frequency regulation. This optimization translates to a more effective utilization of this valuable resource.
    Synergistic Performance Enhancement: The control strategy fosters a synergistic relationship between MEVES and other potential IUMG storage devices, such as FESS and BESS. This collaborative approach unlocks the full potential of the combined storage capacity, leading to a significant improvement in overall IUMG performance and frequency regulation capabilities.

Furthermore, the present research work employs a Evaluation multifaceted approach that strengthens the validity and originality of the findings:

1. **Extensive Time-Domain Simulations:** The proposed controller’s performance is meticulously evaluated through comprehensive time-domain simulations. These simulations incorporate established performance metrics, including Integral of Squared Error (ISE), Integral of Absolute Error (IAE), Integral of Time Multiplied By Squared Error (ITSE), and Integral of Time Multiplied By Absolute Error (ITAE), providing a thorough assessment of the controller’s effectiveness under various operating conditions.
2. **Comparative Analyses with Benchmark Controllers:** The practicality and advantages of the proposed approach are demonstrably highlighted through rigorous comparative analyses with established controllers, such as the 3DOF-PID and PID controllers. This comparative evaluation allows for a clear understanding of the proposed controller’s superior performance in real-world applications.
3. **Cross-Algorithm Comparison for Optimization Validation:** The efficacy of the COA is further validated by comparing its performance against well-known optimizers like WSO and RSA across diverse IUMG operating scenarios. This comparative analysis reinforces the effectiveness of the COA algorithm in identifying optimal controller parameters for various IUMG conditions.
4. **Robustness Testing for Enhanced Reliability:** To ensure the robustness of the proposed COA-based 1PD-3DOF-PID cascade controller, the study incorporates robustness testing. This testing involves simulating parameter fluctuations of ± 25% in selected IUMG parameters, demonstrating the controller’s ability to maintain performance even under varying operating real-world conditions.

These contributions collectively represent significant advancements in the field of LFC within IUMGs, providing innovative insights and practical solutions for enhancing frequency regulation and overall IUMGs performance. Building upon our comprehensive review of the existing literature, this study injects significant novelty into the field of LFC for IUMGs by introducing two paramount contributions. Firstly, the research leverages the 1PD-3DOF-PID controller, marking a groundbreaking approach within the LFC domain. This novel control strategy has not been previously explored for IUMG frequency regulation, offering a fresh perspective for tackling this critical challenge. Secondly, the design of the proposed controller prioritizes the pioneering integration of the COA. By harnessing the COA’s optimization capabilities for LFC design in IUMGs, this work represents the first of its kind in this specific domain. These distinctive contributions underscore the originality and potentially transformative impact of this research on the advancement of IUMG-based LFC strategies.

### Organization of the paper

**Figure 1:**
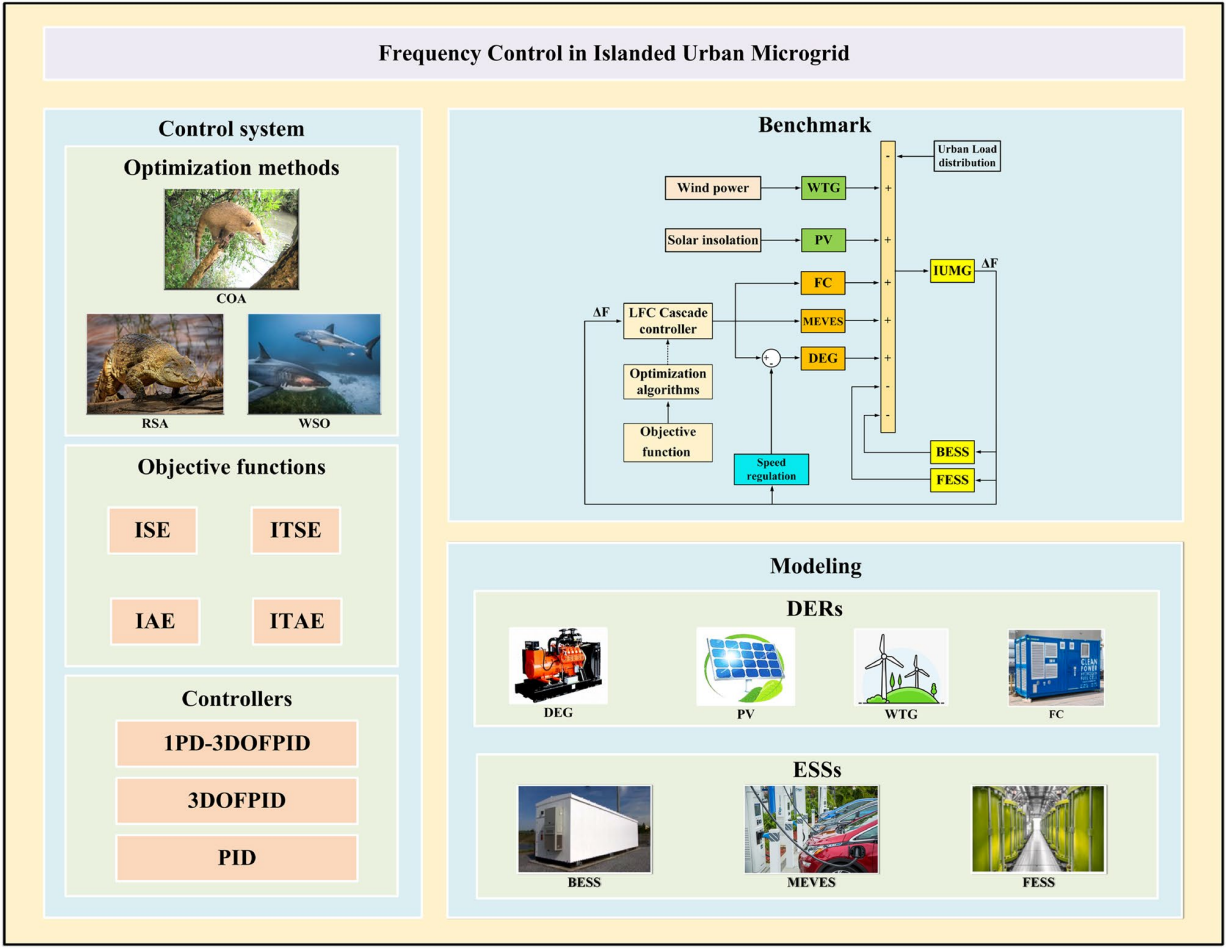
The graphical abstract of the present work.

The remainder of this paper is meticulously organized to facilitate a comprehensive understanding of the proposed advancements: Sections “IUMG: Investigation, Analysis Mathematical Model and Modeling” through “Implementation of the Proposed LFC method: Design and Settings” delve into the intricacies of the IUMG model, the COA optimization approach, a detailed analysis of the objective function, and the layout of the proposed controller alongside its design using optimization algorithms. Subsequently, Sections “Simulated results” and “Discussion and analysis of results” present the simulation results, followed by a rigorous discussion and analysis of these findings. To culminate the work, Sections “Advantages of the proposed approach” through “Suggestions for future research” illuminate the advantages of the proposed approach, draw insightful conclusions from the research, and propose potential avenues for future exploration. For visual clarity and a concise overview of the research structure, Fig. 1 presents a graphical representation of the workflow.

## IUMG: Investigation, analysis mathematical model and modeling

**Figure 2:**
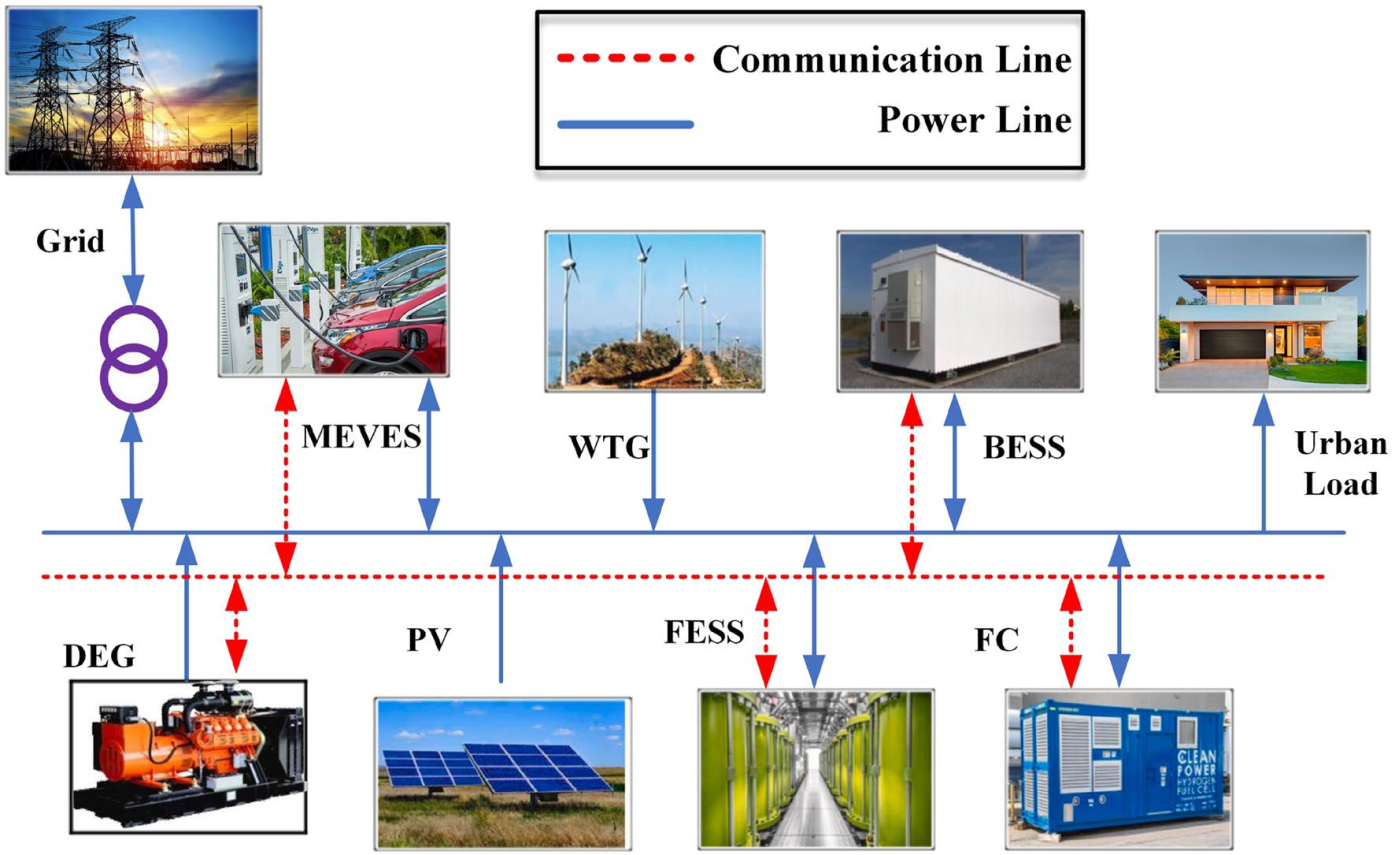
The general schematic of the studied IUMG along with the communication links and the power line.

Figure 2 depicts the proposed IUMG schematic. This schematic illustrates the key components of the IUMG, including load-capable units and bidirectional data exchange facilitated by communication links and power lines.

**Figure 3:**
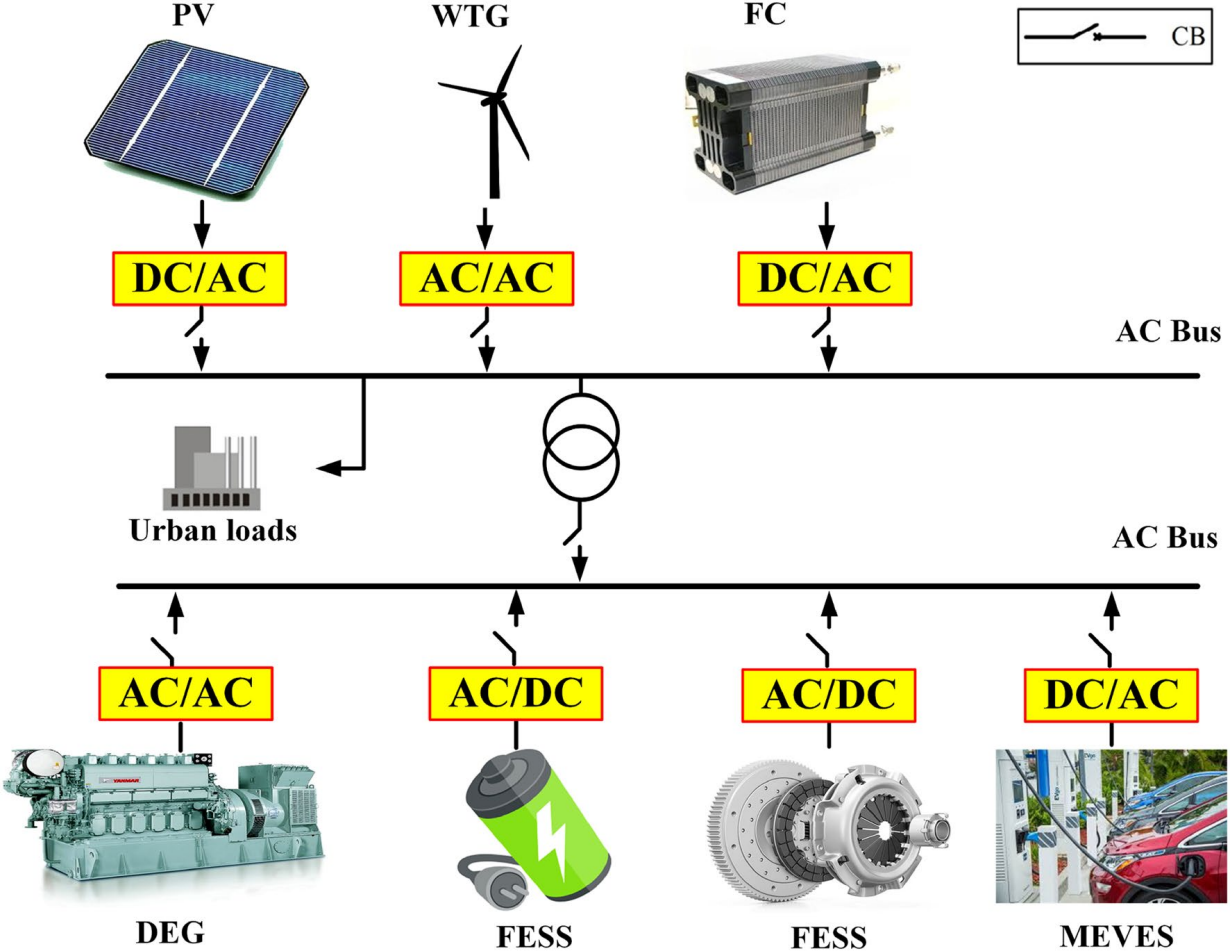
Connecting DG units and resources by means of DC / AC interface inverters to IUMG.

On the other hand, in more detail according to Fig. 3, the IUMG comprises a diverse array of components, including Photovoltaic (PV) systems, Wind Turbine Generators (WTGs), Diesel Engine Generators (DEGs), Fuel Cells (FCs), MEVES units, BESS, FESS, AC buses, Circuit Breakers (CBs), DC-AC and AC-DC converters, and urban loads. Each department within the IUMG is interconnected via inverters and equipped with its own CB for protection on the AC bus during significant disruptions, as referenced in^44^. The IUMG configuration categorizes its constituent units into two primary classifications: dispatchable and non-dispatchable. Dispatchable units, including DEG, FC, and MEVES, can be actively controlled within the secondary loop for LFC purposes. Conversely, non-dispatchable units, such as WTG and PV systems, rely on RES. This dependence on renewable sources results in intermittent and weather-dependent power output, making them unsuitable for direct control within the LFC scheme.

This work simplifies the analysis of frequency response in IUMGs by strategically omitting power electronic converter models. Instead, it leverages linearized first-order transfer functions to represent the dynamic behavior of PV, WTG, DEG, FC, BESS, and FESS subsystems. The rationale behind this simplification lies in the suitability of lower-order models for small signal analysis and frequency control studies. Notably, the research introduces a novel MEVES model specifically designed for the IUMG LFC. Within the IUMG architecture, BESS and FESS function as localized power sources, actively mitigating frequency deviations through microgrid power exchange. This inherent capability establishes them as valuable resources for maintaining grid stability. Furthermore, for RES characterized by slower response times, the additional support of ESS proves beneficial for achieving advanced frequency control.

### WTG model

WTGs are inherently variable power sources due to fluctuations in wind profiles, introducing instability into the grid^66,67^. To account for this variability within the IUMG model, the WTG is represented as a component that injects power fluctuations. The output power (*P*_*wind*_) and mechanical power (*P*_*mech*_) of the WTG are determined by Eqs. (1) and (2) as presented in^68^:

1 $$\begin{array}{*{20}l} {P_{wind} = \eta P_{mech} } \hfill \\ \end{array}$$

2 $$P_{mech} = \frac{1}{2}C_{p} \left( {\lambda ,\beta } \right)A\rho_{a} V_{w}^{3}$$

where *η* and *ρ* are the WTG’s efficiency and air density, respectively; *λ* = *ω*_*r*_*R*/*υ* is tip speed ratio; *ω*_*r*_ is the angular velocity of the rotor and *R* is the blade length. As a natural resource, the resultant power of a wind turbine is fluctuating due to the time-variant wind direction and the wind speed (*V*). The power coefficient, *C*_*p*_, and several other physical parameters are combined to calculate the resultant power of the WTG, as previously mentioned and reiterated in numerous publications. *C*_*p*_, which represents the power-capturing efficiency of the WTG, is influenced by two primary factors: the tip speed ratio (*λ*) and the blade pitch angle (*β*), It is defined as follows:

3 $$C_{p} = 0.5176\left( {\frac{116}{{\lambda_{i} }} - 0.4\beta - 5} \right)e^{{\frac{ - 21}{{\lambda_{i} }}}} + 0.0068\lambda$$

where *λ*_*i*_ satisfies the following Eq. (4):

4 $$\frac{1}{{\lambda_{i} }} = \frac{1}{\lambda + 0.08\beta } - \frac{0.035}{{\beta^{3} + 1}}$$

Among the DER units integrated within the IUMG, the inherent characteristics of WTGs have minimal influence on the IUMG’s frequency response. Therefore, wind power is chosen in this work as an illustrative example of a variable power resource within the IUMG.

**Figure 4:**
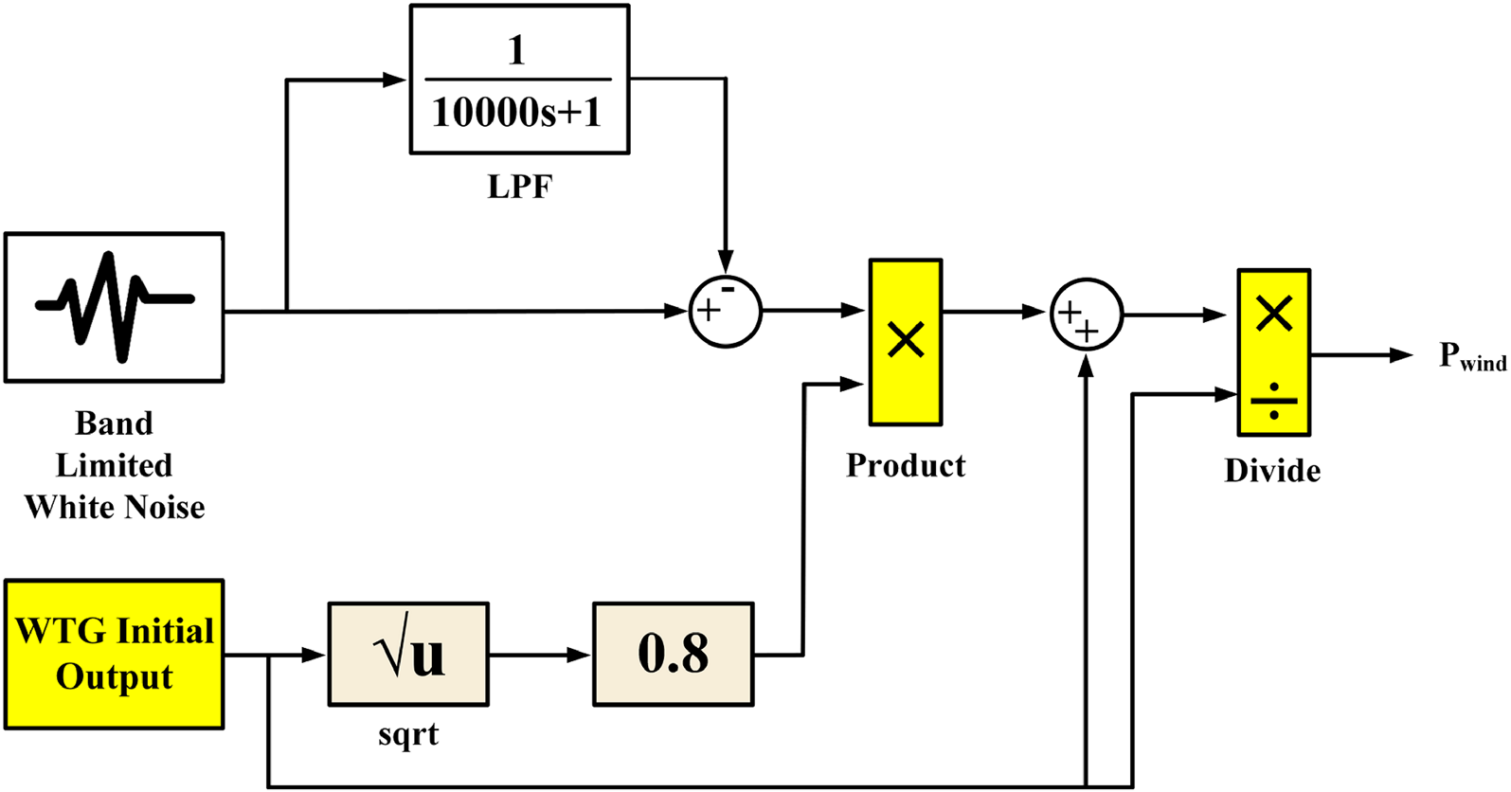
Mathematical models of a practical WTG system.

Figure 4 showcases a mathematical model of a real WTG system for in-depth analysis. For further theoretical background on WTG modeling, refer to^10,69,70^.

While WTGs exhibit inherent non-linear behavior, this work adopts a simplified approach for the sake of tractability. The WTG is represented by a first-order lag transfer function, as detailed in Eq. (5) ^69^. This linearization facilitates analysis of the IUMG’s frequency response characteristics.

5 $$G_{WTG} \left( s \right) = \frac{{\begin{array}{*{20}l} {\Delta P_{WTG} } \hfill \\ \end{array} }}{{P_{wind} }} = \frac{{\begin{array}{*{20}l} {K_{WTG} } \hfill \\ \end{array} }}{{1 + sT_{WTG} }}$$

### PV model

The PV systems are favored for their abundant solar irradiation and ease of installation^71,72^. Calculating their power production relies on two key factors: solar irradiance level and temperature^73–75^. The following equation may be used to determine the resulting power from PV systems:

6 $$P_{PV} = \varphi \cdot S \cdot \xi \cdot \left( {1 - 0.005\left( {T_{A} - 25} \right)} \right)$$

In this context, the efficiency of the PV array is represented by the symbol *φ*, typically falling within the range of 9% to 12%. The effective area the PV panels cover is denoted as *S* and measured in square meters (m^2^). At the same time, solar irradiance is symbolized by *ξ* with units in kilowatts per square meter (kW/m^2^). Furthermore, *T*_*A*_ signifies the ambient temperature, often standardized at 25 °C. Consequently, the power output of the PV system, *P*_*PV*_, is contingent on the solar irradiance level, given that *S* and *ξ* remain constants.

The PV system comprises power electronic interfaces, namely an inverter and an interconnection device, designed for synchronization with the IUMG. The transfer function of the PV system is characterized by a first-order lag, as represented by Eq. (7) ^73^:

7 $$G_{PV} \left( s \right) = \frac{{\begin{array}{*{20}l} {\Delta P_{PV} } \hfill \\ \end{array} }}{{\Phi_{solar} }} = \frac{{\begin{array}{*{20}l} {K_{PV} } \hfill \\ \end{array} }}{{1 + sT_{PV} }}$$

**Figure 5:**
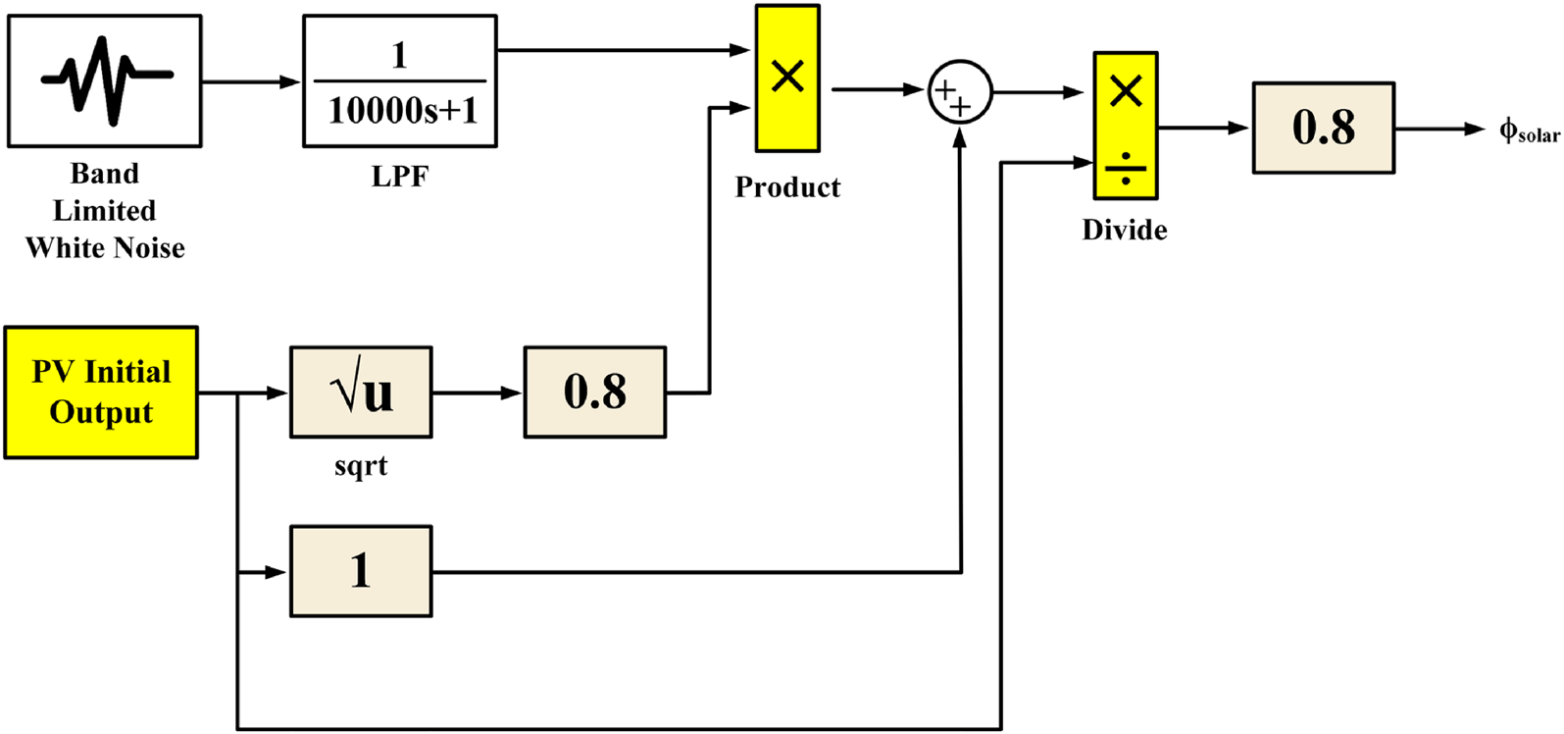
Mathematical models of a practical PV system.

Figure 5 presents a mathematical model of a real-world PV system^69^.

### DEG model

**Figure 6:**
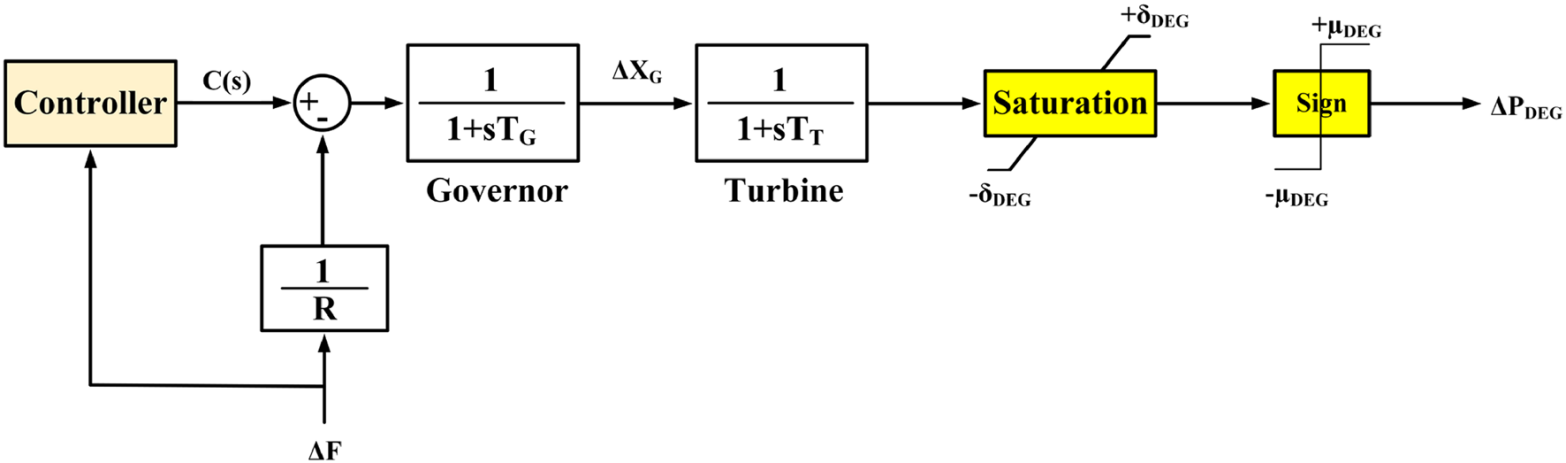
Mathematical model of a DEG.

The DEG is a compact, efficient power generation unit with quick startup and responsive output control, ensuring uninterrupted, high-quality power to critical loads on diesel-electric generators. The utilization of a DEG underscores its operational principle^76^. The DEG’s mathematical model, comprising a governor and a turbine, is visually represented in Fig. 6^77^. Equation (8) presents a simplified transfer function model for the DEG:

8 $$G_{DEG} \left( s \right) = \frac{{\begin{array}{*{20}l} {\Delta P_{DEG} } \hfill \\ \end{array} }}{C\left( s \right)} = \left( {\frac{{\begin{array}{*{20}l} 1 \hfill \\ \end{array} }}{{1 + sT_{G} }}} \right)\left( {\frac{1}{{1 + sT_{T} }}} \right)$$

In Fig. 6, the symbols *ΔF* and *C(s)* denote the frequency deviation and the command signal from the LFC output controller, respectively. *T*_*G*_ represents the governor’s time constant, while *T*_*T*_ stands for the time constant of the diesel generator. *ΔX*_*G*_ signifies the governor’s valve speed control coefficient of the DEG, denoted as *R*. Power increase and ramp rate constraints are represented by ± *μ*_*DEG*_ and ± *δ*_*DEG*_, respectively. *ΔP*_*DEG*_ indicates the DEG’s power output. A *ΔP*_*DEG*_ value of zero signifies a power equilibrium between demand and generation, requiring no adjustments. Conversely, a positive *ΔP*_*DEG*_ value indicates a greater demand than actual power generation, while a negative *ΔP*_*DEG*_ value implies insufficient actual power relative to demand^78^. Further details about the DEG model can be referenced in^77,79^.

### Non-mobile ESS model

Non-mobile ESS units like BESS and FESS are essential for system stability, providing quick power and promoting renewable energy shift from fossil fuels. BESS uses electrochemical principles, while FESS stores electrical energy as kinetic energy with minimal frictional losses^80–82^. The transfer function of both BESS and FESS can be represented by a first-order lag transfer function, as given in Eqs. (9) and (10), respectively^73^:

9 $$G_{BESS} (s) = \frac{{\Delta P_{BESS} }}{\Delta F} = \frac{1}{{1 + sT_{BESS} }}$$

10 $$G_{FESS} (s) = \frac{{\Delta P_{FESS} }}{\Delta F} = \frac{1}{{1 + sT_{FESS} }}$$

### Fuel cell model

FCs efficiently generate electricity from hydrogen or other fuels, producing electricity, water, and heat. Integrating FCs in MGs improves performance and promotes hydrogen energy utilization. This paper integrates FC with non-mobile ESSs to counteract system instability caused by WTG and PV intermittency. An inverter and interconnection device follow the FC block. The FC’s transfer function can be expressed as a first-order lag in Eq. (11) ^73^:

11 $$G_{FC} (s) = \frac{{\Delta P_{FC} }}{r} = \frac{1}{{1 + sT_{FC} }}$$

### Mobile ESS (MEVES)

The battery pack within a MEVES unit functions as a large-scale BESS^83^. Vehicle-to-Grid (V2G) technology facilitates the integration of these mobile BESS units into LFC analysis^84^. By regulating the charging and discharging cycles of MEVES units in accordance with the central load dispatching center’s LFC signal (*C*(*s*)), precise frequency management can be achieved^10,85–87^.

**Figure 7:**
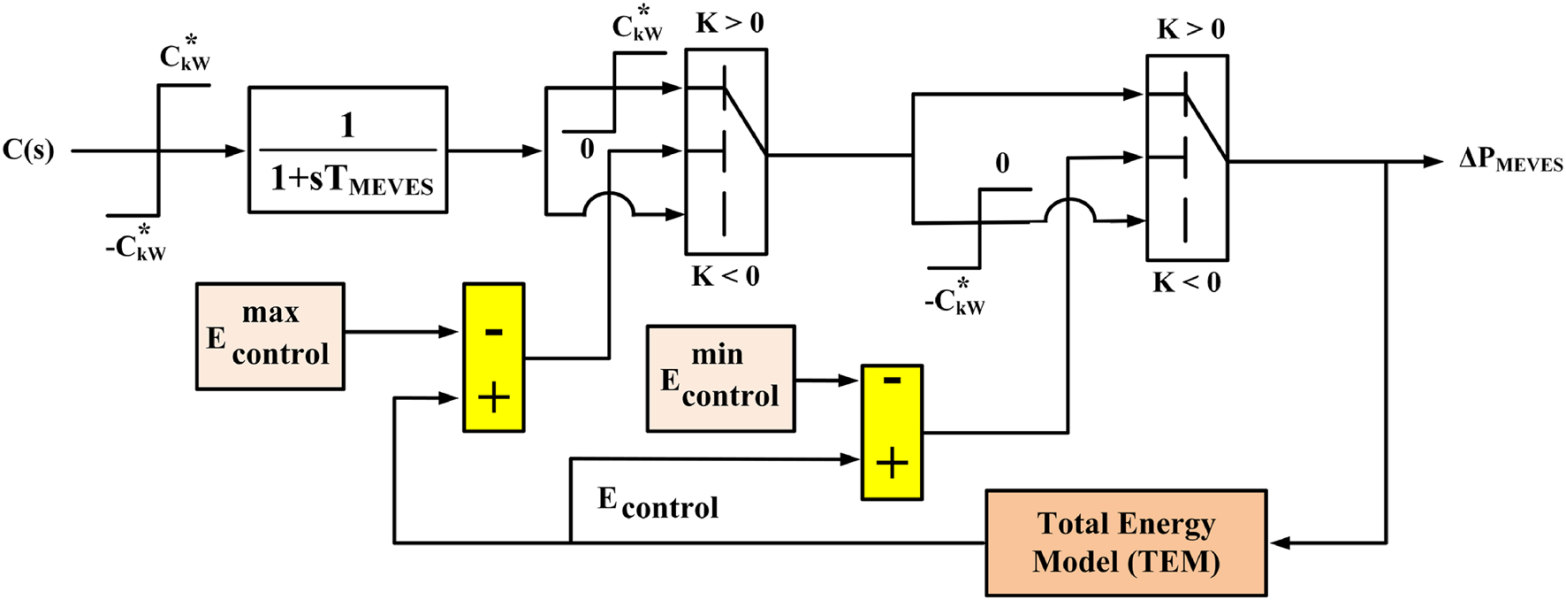
Mathematical model of a MEVES.

This work adopts the MEVES modeling approach presented in^10,54^, which itself builds upon the foundational work in^85,86,88,89^. Figure 7 depicts the detailed model of a MEVES unit. Notably, the model takes the controller’s output signal, denoted as *C*(*s*), as its input, as illustrated in Fig. 7. *ΔP*_*MEVES*_ represents the total power output of the MEVES unit, signifying either battery charging or discharging.

The presented MEVES model (see Fig. 7) readily facilitates the determination of their charging or discharging state under steady-state conditions. The responsiveness of the LFC signal can be modulated by controlling the number of participating MEVES units while considering user convenience, as reflected by their State of Charge (SOC)^85,86^. It is crucial to emphasize that MEVES participation in the V2G control system adheres to user-centric battery management strategies^90^. Battery charging is limited to 85% of capacity, followed by operation within a restricted range of 85–5% SOC. This range prioritizes user convenience for subsequent trips. Furthermore, to extend battery lifespan, which can be compromised at full charge (100% SOC), the maximum allowable SOC is capped at 90%^60,88^. The lower limit of 5% SOC ensures sufficient reserve power for users to complete planned journeys^91^.

In this work, it is posited that MEVES units with a SoC of 80% or higher remain uncontrolled, rendering them ineligible for participation in the V2G control scheme. Following the charging process, MEVES can only respond to the LFC signal within the confines of their energy capacity, denoted as the MWh limit, as specified by Eq. (12) ^85,86,92^.

12 $$E_{control}^{\min } \le E_{control} \le E_{control}^{\max }$$

where *E*_*control*_ is the total energy of the MEVES that can be controlled, $E_{control}^{\max }$ and $E_{control}^{\min }$ are the maximum and lower energy capacity limits, respectively, and *E*_*control*_ is the total energy of the controllable MEVES. The aforementioned energy capacity limitations are determined using Eqs. (13) and (14), which are dependent on the control method, also known as the stated SOC.

13 $$E_{control}^{\min } = \frac{{N_{control} \cdot C_{kWh}^{*} }}{1000} \times 0.8$$

14 $$E_{control}^{\max } = \frac{{N_{control} \cdot C_{kWh}^{*} }}{1000} \times 0.9$$

**Figure 8:**
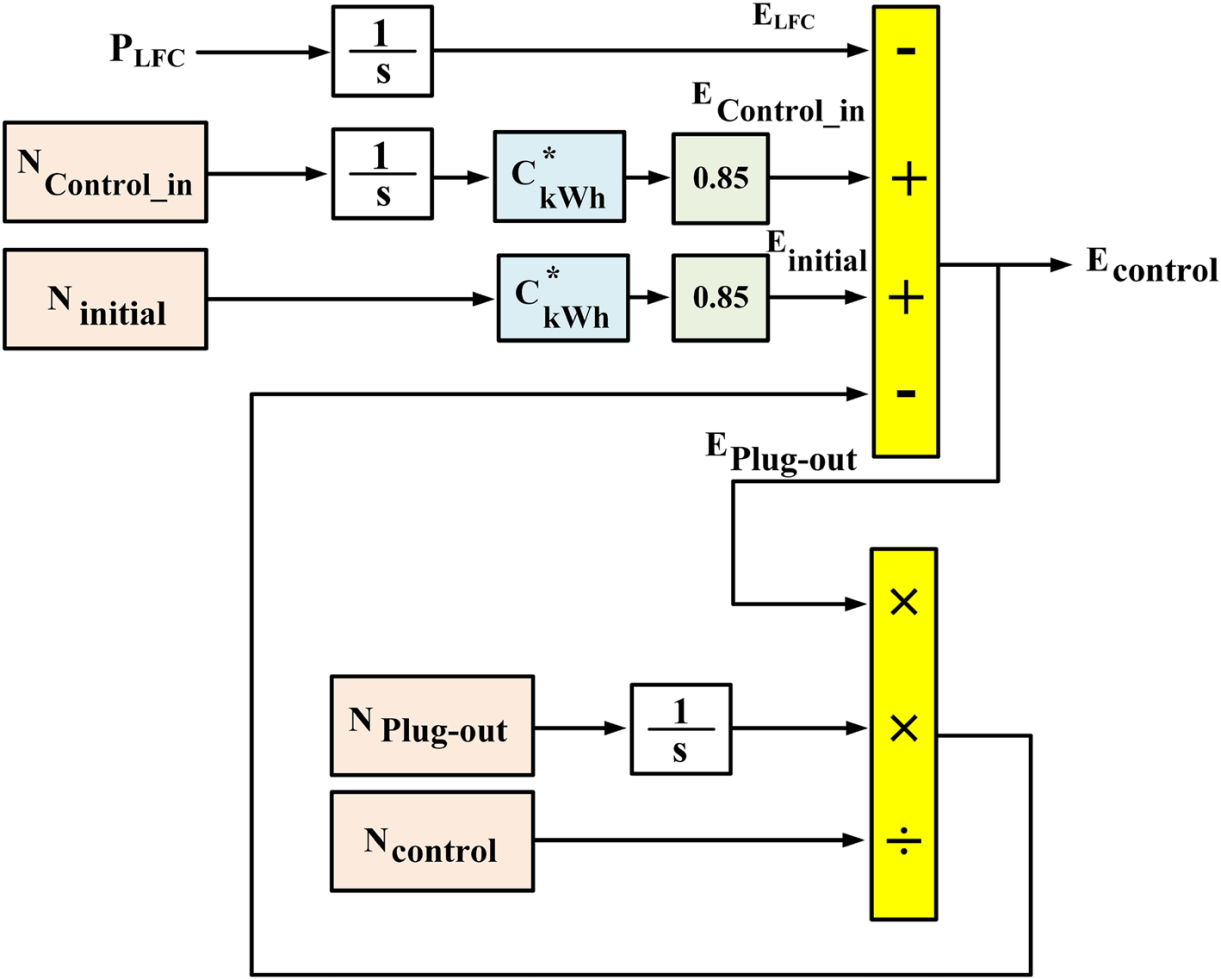
Mathematical model of a TEM in MEVES model.

The comprehensive energy model, referred to as Total Energy Model (TEM) and depicted in Fig. 7, is illustrated in more detail in Fig. 8. This model yields *E*_*control*_, representing the collective stored energy of all managed MEVES units. Formulations for determining the quantity of managed MEVES units (*N*_*control*_) participating in power exchange with the IUMG are as follows:

15 $$N_{control} = N_{initial} + N_{control\_in} - N_{plug\_out}$$

In the given equations, *N*_*initial*_ represents the initial count of controllable EVs, *N*_*control_in*_ signifies the quantity of MEVESs transitioning from the charging state to the controllable state, and *N*_*plug_out*_ indicates the number of MEVESs transitioning from the charging state to the driving state.

The inverter capacity of the MEVES energy storage system is denoted by $C_{kW}^{*}$; hence, the MEVES can only be charged within a range that is less than or equal to $\pm C_{kW}^{*}$.

Detailed values corresponding to these variables are provided in Table 2 for reference and application Also for more details about the MEVES model, may refer to^10,54,85,86,88^.

**Table 2:** Symbols used in IUMG and their meanings.

Symbol	Value	Unit	Symbol	Value	Unit	Value	Symbol	Unit
*D*	0.012	pu/s	*K*_*PV*_	1	–	*T*_*BESS*_	0.1	s
*M*	0.2	pu/Hz	*T*_*PV*_	1.8	s	*T*_*FC*_	0.28	s
*K*_*WTG*_	1	–	*R*	2.4	pu/s	*T*_*MEVES*_	4	s
*T*_*WTG*_	1.5	s	*T*_*G*_	0.08	s	C*_kW_	3c	kW
*T*_*T*_	0.1	s	*T*_*IC*_	0.004	s	C*_kWh_	15	kWh
*δ*_*DEG*_	0.01	pu/s	*μ*_*DEG*_	0.025	pu	*N*_*control*_	82	N
*T*_*IN*_	0.04	s	*T*_*FESS*_	0.1	s	*N*_*initial*_	90	N
*N*_*control_in*_	12	N	*N*_*plug_out*_	20	N	*f*	50	Hz

Remak_ pu: perunit, Hz: Hertz, s: Second, N: Number.

### Modeling of IUMG

A comprehensive body of research has explored diverse and dynamic modeling approaches for MGs to evaluate frequency regulation effectiveness^10,11,78,93–102^. Building upon this foundation, this work presents Eq. (16) employed to assess LFC within the IUMG after integration with various Distributed Energy Resources (DERs). These DERs encompass RES such as PV and WTGs, mobile ESS like MEVES, and non-mobile ESS solutions including BESS and FESS. Additionally, other DERs like FC, also DEG are incorporated into the IUMG model.

16 $$\Delta P_{WTG} + \Delta P_{PV} + \Delta P_{FC} + \Delta P_{DEG} + \Delta P_{EV} - \Delta P_{BESS} - \Delta P_{FESS} - \Delta PD = \left( {D + sM} \right)\Delta F$$

**Figure 9:**
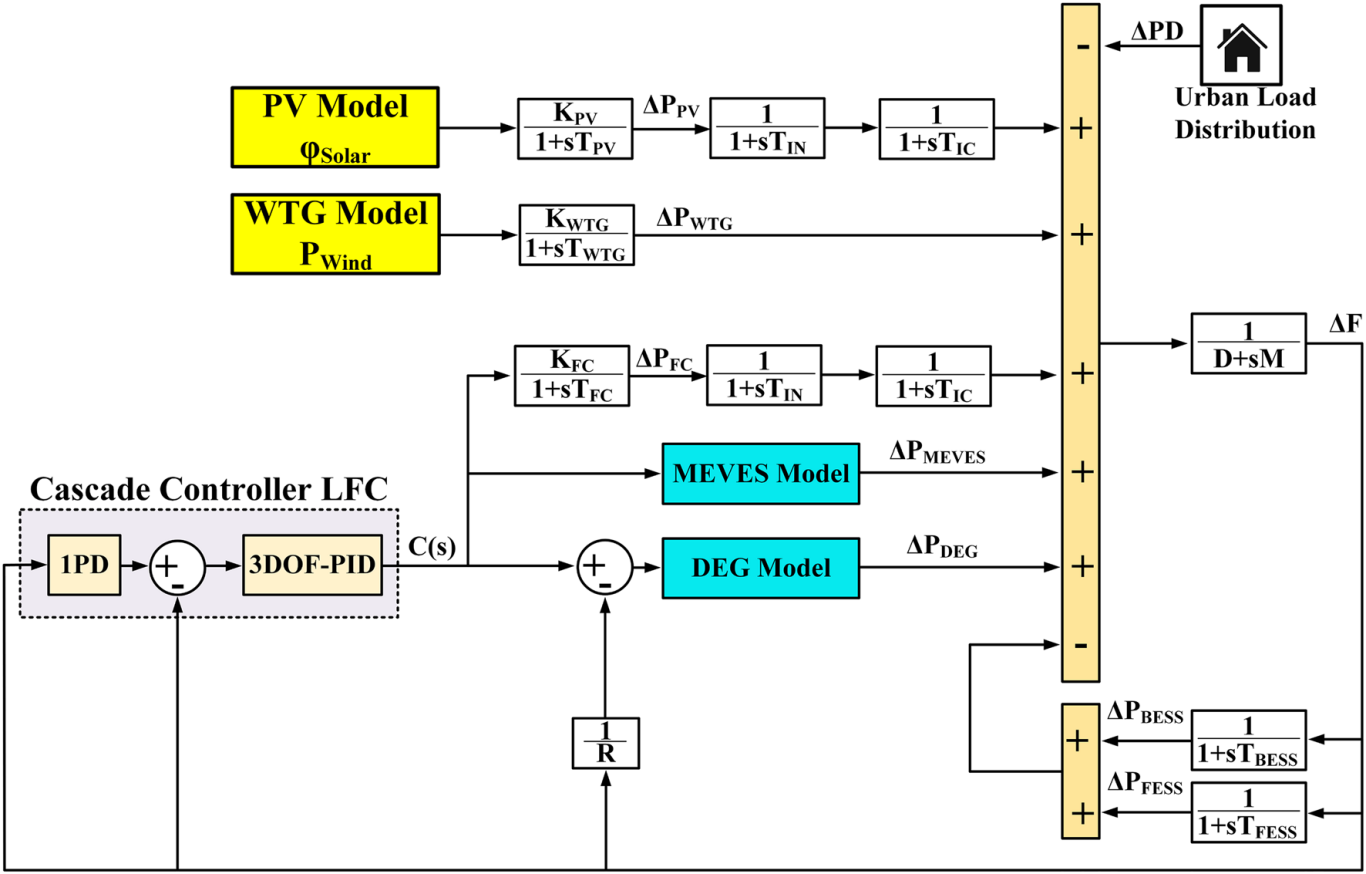
The studied IUMG model.

The charging operations of these units are indicated by the “−” symbol placed before *ΔP*_*BESS*_ and *ΔP*_*FESS*_ in Eq. (16). Figure 9 depicts the investigated IUMG model. Additionally, Table 2 provides a comprehensive list of symbols employed within the IUMG model, along with their corresponding definitions.

#### Remark

The MEVES units play a pivotal role in ensuring system stability by dynamically supplying and absorbing electricity within constrained timeframes. To address the specific charging characteristics of MEVES and minimize data storage requirements in LFC system design, this work proposes a novel approach for modeling MEVES dynamics. This approach leverages the latest advancements in MEVES modeling, detailed in the subsequent section.

Furthermore, to facilitate rapid active power generation, a model for DEGs is presented. Additionally, a dynamic model for WTG and PV systems is introduced. This model is strategically de-loaded to create ample headroom for these RESs to effectively participate in the IUMG’s frequency regulation scheme, ensuring seamless collaboration with other generation units.

## Introduction of COA

The field of LFC has witnessed a growing interest in metaheuristic optimization algorithms to address limitations encountered with traditional methods. This surge in attention stems from the realization, underscored by the No-Free-Lunch Theorem, that a single, universally effective optimization algorithm is not achievable^103^. This theorem motivates researchers to continually develop new algorithms for tackling diverse optimization problems^104^. Recent advancements in this domain have yielded several promising metaheuristic algorithms, including the Mountaineering Team-Based Optimization (MTBO)^105^, Corona-Virus Search Optimizer (CVSO)^106^, Turbulent Flow of Water-based Optimization (TFWO)^107^. These algorithms offer the potential to overcome limitations associated with existing techniques and enhance the efficacy of LFC strategies. All the aforementioned metaheuristic optimization algorithms share the common characteristics of exploration and exploitation. However, their distinctiveness lies in the unique biological concepts that inspire their optimization processes^108^. Existing research has demonstrated the effectiveness of these methods in addressing LFC challenges.

This work investigates the application of three specific algorithms –WSO, RSA, and COA – for optimizing controller gains within the LFC framework of IUMG. The WSO draws inspiration from the sophisticated echolocation capabilities employed by great white sharks during hunting^63^. Similarly, the RSA mimics the cooperative hunting behavior exhibited by crocodiles^62^. COA, on the other hand, is inspired by the diverse natural behaviors of coatis, including their interactions with iguanas and their strategies for evading predators^61^. Notably, the COA technique offers a straightforward and efficient optimization approach for LFC applications, without requiring adjustments to control parameters. Capitalizing on the aforementioned advantages of the COA, this work adopts it as the dominant optimizer. The WSO and RSA, which serve as evaluator algorithms. The combined application of these three optimizers demonstrably addresses the LFC challenge within the proposed IUMG framework.

### Inspiration

**Figure 10:**
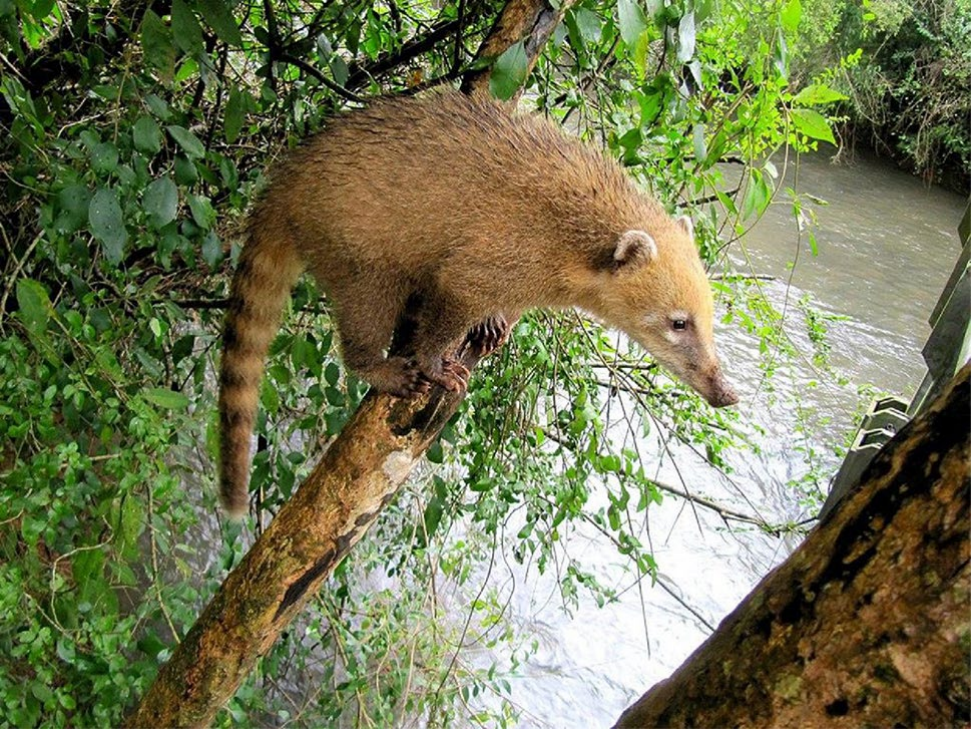
Picture of a coati while hunting^61^.

Figure 10 presents coatis, omnivorous mammals native to the Americas^37^. The design of the COA draws inspiration from their cooperative group hunting behaviors, particularly when targeting prey such as iguanas. This collaborative approach informs the COA’s ability to adapt and prevent convergence towards suboptimal solutions during the optimization process. The algorithm leverages cooperative exploration to achieve comprehensive search space coverage and utilizes an adaptive mechanism inspired by coati hunting tactics to avoid becoming trapped in local optima. This bio-inspired design underpins the COA’s effectiveness in tackling complex optimization problems^61^.

### Algorithm execution process: exploration and exploitation

The COA, similar to many optimization algorithms, operates in two fundamental stages: exploration and exploitation. In essence, COA leverages these two complementary phases–exploration inspired by coati hunting and exploitation inspired by coati evasion – to achieve efficient search and optimal solution identification.

#### Exploration phase: mimicking coati hunting strategy

The exploration phase in COA draws inspiration from the cooperative hunting behavior of coatis targeting prey like iguanas. Here, the algorithm emphasizes searching for new and potentially superior regions within the search space. This is mathematically modeled by simulating two coati sub-populations (i.e. Climbing Coatis and Waiting Coatis).

#### Exploitation phase: mimicking coati hunting strategy

The exploitation phase is inspired by the natural behavior of coatis when escaping predators. Here, the algorithm focuses on refining promising areas identified during the exploration phase. Mathematically, this is modeled by simulating a coati’s escape maneuver.

### Advantages and features

The COA presents itself as a compelling optimization tool for addressing global optimization problems. It boasts several key advantages^61^:

1. **Parameter-free design**: A defining strength of COA resides in its parameter-free structure. This eliminates the need for manual parameter tuning, streamlining algorithm implementation and mitigating the potential for user bias.
2. **Broad applicability:** COA demonstrates remarkable efficiency in tackling optimization problems across diverse scientific disciplines. This efficacy extends to complex, high-dimensional problems frequently encountered in real-world applications.
3. **Balanced exploration and exploitation:** COA excels in achieving a well-balanced approach to exploration and exploitation during the search process. This characteristic enables it to converge rapidly towards suitable decision variable values in optimization tasks, particularly for problems exhibiting complexity.
4. **Real-world performance:** COA exhibits robust performance when managing real-world optimization applications. This translates to effective solutions for practical engineering challenges.

Extensive studies and reviews have positioned COA favorably against established optimization algorithms. Benchmarking has been conducted against eleven well-regarded algorithms, including Multi-Verse Optimizer (MVO), WSO, Gravitational Search Algorithm (GSA), Marine Predators Algorithm (MPA), Particle Swarm Optimization (PSO), Genetic Algorithm (GA), Tunicate Search Algorithm (TSA), Gray Wolf Optimization (GWO), Whale Optimization Algorithm (WOA), and Teaching–Learning Based Optimization (TLBO)^61^. Consistently superior performance across these comparisons underscores the strength and promise of COA.

In conclusion, the aforementioned advantages establish COA as a powerful new contender in the realm of optimization tools. Its parameter-free design, broad applicability, balanced exploration–exploitation capabilities, and real-world effectiveness make it a strong candidate for tackling complex optimization problems, including the LFC challenge within IUMGs.

### The procedure of initializing the algorithm

COA technique is a population-based metaheuristic analyzing coatis, determining decision variables based on their spatial location within a search space, seeded randomly using Eq. (17) ^61^.

17 $$\begin{gathered} X_{i} :x_{i,j} = lb_{j} + r \cdot \left( {ub_{j} - lb_{j} } \right) \hfill \\ i = 1,2,...,N \hfill \\ j = 1,2,...,m \hfill \\ \end{gathered}$$

In this context, *X*_*i*_ represents the position of the *ith* coati within the search space, *x*_*i,j*_ denotes the value of the *jth* decision variable, *N* stands for the total number of coatis, *m* indicates the number of decision variables, r is a random real number within the [0, 1] interval, and *lb*_*j*_ and ubj correspond to the lower and upper bounds of the *jth* decision variable, respectively^61^. Metaheuristic algorithms like COA visualize coati populations and evaluate candidate solutions’ quality using matrix representations, with an objective function selecting the best member.

### Mathematical representation of the COA

The COA procedure for relocating coatis models natural behaviors like pursuing iguanas and evading predators, presenting the population in two phases, focusing on strategies and tactics.

#### Phase 1: exploration involves hunting iguanas and employing attacking tactics

Coati uses behavior simulation to attack iguanas, showcasing global exploration and problem-solving through tree climbing and descent anticipation, using Eq. (18) for mathematical simulation.

18 $$\begin{gathered} X_{j}^{P1} :x_{i,j}^{P1} = x_{i,j} + r \cdot (Iguana_{j} - I \cdot x_{i,j} ) \hfill \\ i = 1,2,...,\left\lfloor \frac{N}{2} \right\rfloor \hfill \\ j = 1,2,...,m \hfill \\ \end{gathered}$$

Iguana is randomly placed in search space, followed by coatis, who approximate movements using Eq. (19) and (20) based on assigned positions.

19 $$\begin{gathered} Iguana^{G} :Iguana_{j}^{G} = lb_{j} + r\left( {ub_{j} - lb_{j} } \right) \hfill \\ j = 1,2,...,m \hfill \\ \end{gathered}$$

20 $$\begin{gathered} X_{i}^{P1} :x_{i,j}^{P1} = \left\{ {\begin{array}{*{20}c} {\begin{array}{*{20}c} {x_{i,j} + r \cdot \left( {Iguana_{j}^{G} - I \cdot x_{i,j} } \right),} & {F_{{Iguana^{G} }} < F_{i} } \\ \end{array} } \\ {\begin{array}{*{20}c} {x_{i,j} + r\left( {x_{i,j} - Iguana_{j}^{G} } \right),} & {else} \\ \end{array} } \\ \end{array} } \right. \hfill \\ for\,i = \left\lfloor \frac{N}{2} \right\rfloor + 1,\left\lfloor \frac{N}{2} \right\rfloor + 2,...,N \hfill \\ j = 1,2,...,m \hfill \\ \end{gathered}$$

Updated coatis can be adopted if new location improves objective function value, while previous positions are retained. This update condition applies to *i* = 1, 2,…, N when Eq. (21) is used for simulation.

21 $$X_{i} = \left\{ {\begin{array}{*{20}c} {\begin{array}{*{20}c} {X_{i}^{P1} ,} & {F_{i}^{P1} < F_{i} } \\ \end{array} } \\ {\begin{array}{*{20}c} {X_{i} ,} & {else} \\ \end{array} } \\ \end{array} } \right.$$

Here The new location that has been computed for the ith coati is $X_{i}^{P1}$, $x_{i,j}^{P1}$ is its *j*th dimension, $F_{i}^{P1}$ is its objective function value, *r* is a real number chosen randomly from the range [0, 1], *Iguana* is a symbol that stands for the iguana’s position in the search area, which more specifically relates to the position of the best member, *Iguana*_*j*_ is its *j*th dimension, *I* is an integer, which is randomly selected from the set {1, 2}, *Iguana*^*G*^ represents the location of the iguana on the ground, which is determined in a random fashion, $Iguana_{j}^{G}$ is its *j*th dimension, $F_{{Iguana^{G} }}$ is its value of the objective function, And the floor function (also known as the greatest integer function) am represented by the symbol “⌊·⌋”.

#### Phase 2: exploitation phase (escaping predators)

Coats in second phase exploit local opportunities using instinctive flee and strategic moves, updating search locations based on natural behavior. Replicate behavior by generating random coati positions near current location using Eqs. (22) and (24).

22 $$\begin{gathered} lb_{j}^{local} = \frac{{{\text{l}} b_{j} }}{t},ub_{j}^{local} = \frac{{ub_{j} }}{t} \hfill \\ t = 1,2,...,T \hfill \\ \end{gathered}$$

23 $$\begin{gathered} X_{i}^{P2} :x_{i,j}^{P2} = x_{i,j} + (1 - 2r) \cdot \left( {lb_{j}^{local} + r \cdot \left( {ub_{j}^{local} - lb_{j}^{local} } \right)} \right) \hfill \\ i = 1,2,...,N \hfill \\ j = 1,2,...,m \hfill \\ \end{gathered}$$

Valid location improved by enhancing objective function value using Eq. (24), indicating significant improvement.

24 $$X_{i} = \left\{ {\begin{array}{*{20}c} {X_{i}^{P2} ,} & {F_{i}^{P2} < F_{i} } \\ {X_{i} ,} & {else} \\ \end{array} } \right.$$

Here Based on the second step of the COA calculation, the new location for the ith coati has been determined to be $X_{i}^{P2}$, $x_{i,j}^{P2}$ is its jth dimension, $F_{i}^{P2}$ is its objective function value, *r* is a random number in the range [0, 1], *t* is the iteration counter, and t is the iteration counter , $lb_{j}^{local}$ and $ub_{j}^{local}$ are the local lower bound and local upper bound of the *j*th decision variable respectively, *lb*_*j*_ and ubj are the lower bound and upper bound of the *j*th decision variable, respectively.

The new location that has been computed for the ith coati is $X_{i}^{P1}$, $x_{i,j}^{P1}$ is its *j*th dimension, $F_{i}^{P1}$ is its objective function value, *r* is a real number chosen randomly from the range [0, 1], *Iguana* is a symbol that stands for the iguana’s position in the search area, which more specifically relates to the position of the best member, *Iguana*_*j*_ is its *j*th dimension, *I* is an integer, which is randomly selected from the set {1, 2}, *Iguana*^*G*^ represents the location of the iguana on the ground, which is determined in a random fashion, $Iguana_{j}^{G}$ is its *j*th dimension, $F_{{Iguana^{G} }}$ is its value of the objective function, And the "floor" function (also known as the "greatest integer" function) am represented by the symbol ⌊·⌋.

### COA cycle flowchart

**Figure 11:**
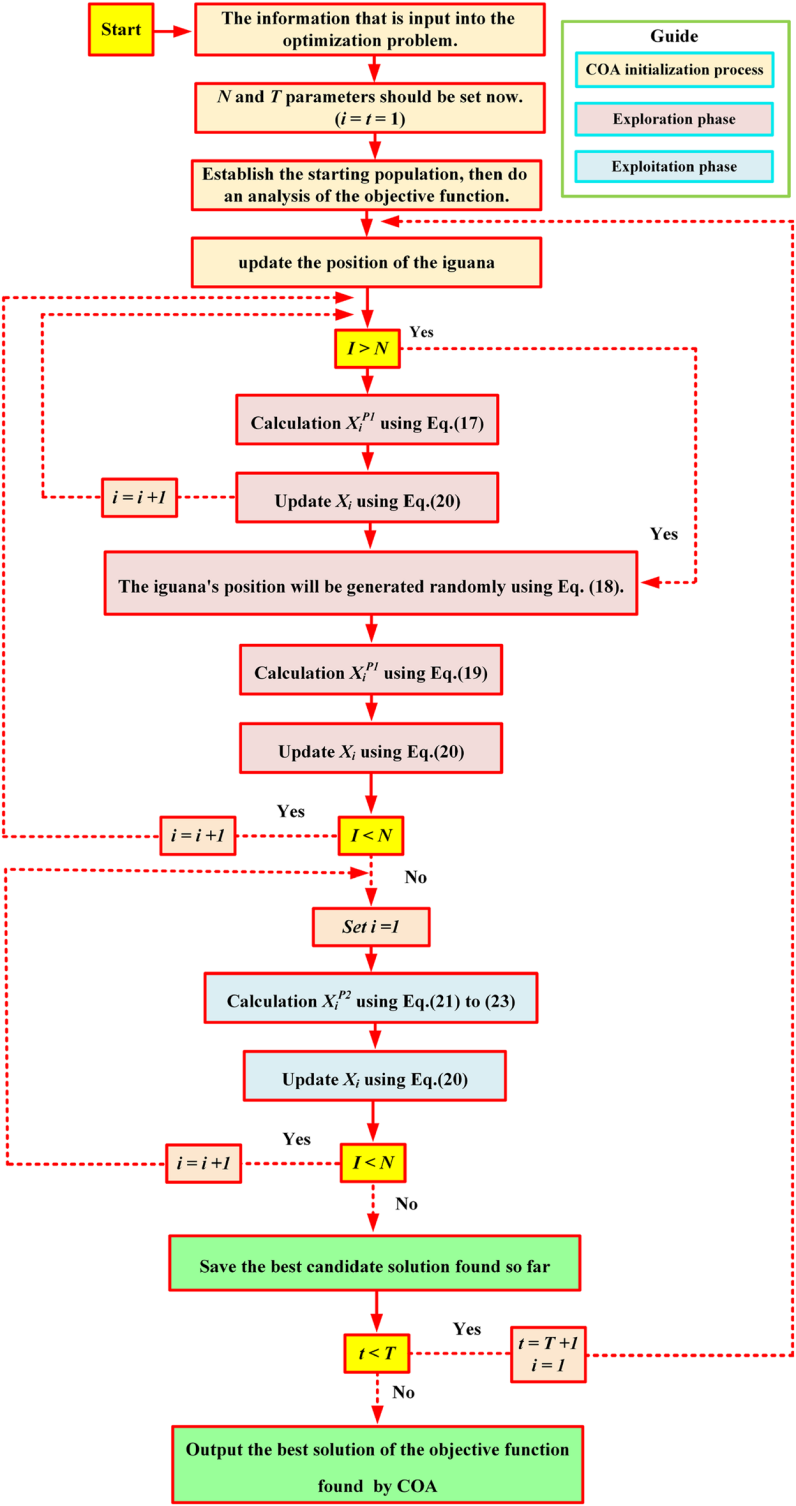
Flowchart of the COA implementation.

COA iterates until the final iteration, adjusting coatis’ positions based on initial stages. The population update procedure continues until the algorithm’s last iteration, achieving the best solution. A flowchart (see Fig. 11) representation are provided.

### Justification for choosing COA as an optimizer

The proposed LFC problem within an IUMG demands an optimization algorithm that excels in two crucial aspects: adaptability and comprehensive search space exploration. Compared to other algorithms like RSA and WSO, the COA algorithm emerges as the dominant choice due to its inherent strengths that effectively address these critical requirements. In essence, the bio-inspired design of the COA algorithm, characterized by its adaptability and ability to explore the entire search space, positions it as the optimal choice for the proposed LFC problem. This balanced approach to exploration and exploitation surpasses the capabilities of the evaluated algorithms, leading to a more efficient and effective search for the optimal solution in optimizing the proposed LFC control scheme within the IUMG.

## Objective function

Effective controller design prioritizes an optimal temporal response in LFC systems. This translates to achieving a response characterized by rapid rise time, minimal overshoot, and a brief settling period. Within the realm of LFC mechanisms, meta-heuristic optimization techniques have emerged as prominent tools for addressing optimization problems specific to these systems, as evidenced by prior research^109,110^. Optimization algorithms play a crucial role in refining controller parameters. These algorithms operate by minimizing a pre-defined performance index, ensuring alignment with the desired response characteristics. Notably, optimizing controller parameters hinges on establishing an objective function that the optimization algorithm aims to minimize.

This work presents a novel approach focused on LFC of IUMGs subjected to external disturbances using evaluation functions based on integral time absolute error. The proposed approach leverages four integral performance indices (Eqs. 25–28): ISE, IAE, ITSE, and ITAE. The numerical values obtained from these objective functions will be critically evaluated and compared to assess the overall performance of the designed controller.

25 $$ISE = \int_{0}^{{t_{sim} }} {(\Delta F)^{2} .dt}$$

26 $$ITSE = \int_{0}^{{t_{sim} }} {t.(\Delta F)^{2} .dt}$$

27 $$IAE = \int_{0}^{{t_{sim} }} {|\Delta F|.dt}$$

28 $$ITAE = \int_{0}^{{t_{sim} }} {t.|\Delta F|.dt}$$

## 1PD-3DOF-PID cascade controller

### Cascade control approach

**Figure 12:**
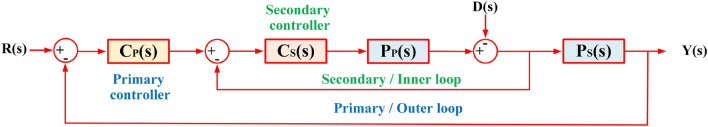
Cascade control conceptionr.

Cascade control regulates two sequential processes, with the inner process’s output serving as input for the outer process. Cascade control is a superior method for managing disturbances in single-loop feedback systems by combining inner and outer loops (see Fig. 12). The outer loop oversees output quality, while the inner loop mitigates supply or internal disturbances. The inner loop maintains control over the output, rejecting disturbances before they propagate throughout the system^111^.

Calculating the system’s output *Y(s)* using Eq. (29) gives the results shown in Fig. 12:

29 $$\begin{aligned} Y\left( s \right) = & \left[ {\frac{{P_{P} \left( s \right)P_{S} \left( s \right)G_{P} \left( s \right)G_{S} \left( s \right)}}{{1 + P_{P} \left( s \right)G_{S} \left( s \right) + P_{P} \left( s \right)P_{S} \left( s \right)G_{P} \left( s \right)G_{S} \left( s \right)}}} \right]R\left( s \right) \\ & - \left[ {\frac{{P_{S} \left( s \right)}}{{1 + P_{P} \left( s \right)G_{S} \left( s \right) + P_{P} \left( s \right)P_{S} \left( s \right)G_{P} \left( s \right)G_{S} \left( s \right)}}} \right]D\left( s \right) \\ \end{aligned}$$

The system receives inputs from *R(s)*, and *D(s)*, with *G*_*P*_*(s)*, *G*_*S*_*(s)*, *P*_*P*_*(s)*, and *P*_*S*_*(s)* denoting the transfer functions of the primary, secondary, inner, and outer loops.

### 1PD-3DOF-PID controller inspiration

LFC controllers reduce step load disturbances, while classical controllers like PI or PID offer reliability and simplicity. Classical controllers struggle with IUMG frequency management, while cascade controllers improve frequency deviation mitigation. Higher DoF and cascade controllers increase complexity and prevent uncontrolled oscillations.

#### 1PD controller

This work introduces a primary controller known as the 1PD (1 + proportional + derivative) controller. Equation (30) represents the transfer functions associated with the primary controller.

30 $$G_{P} (s) = 1 + K_{P} + \frac{{K_{I} }}{s}$$

where for the primary controller’s, *K*_*P*_ and *K*_*I*_ are proportional and integral gains, respectively.

#### 3DOF-PID controller

**Figure 13:**
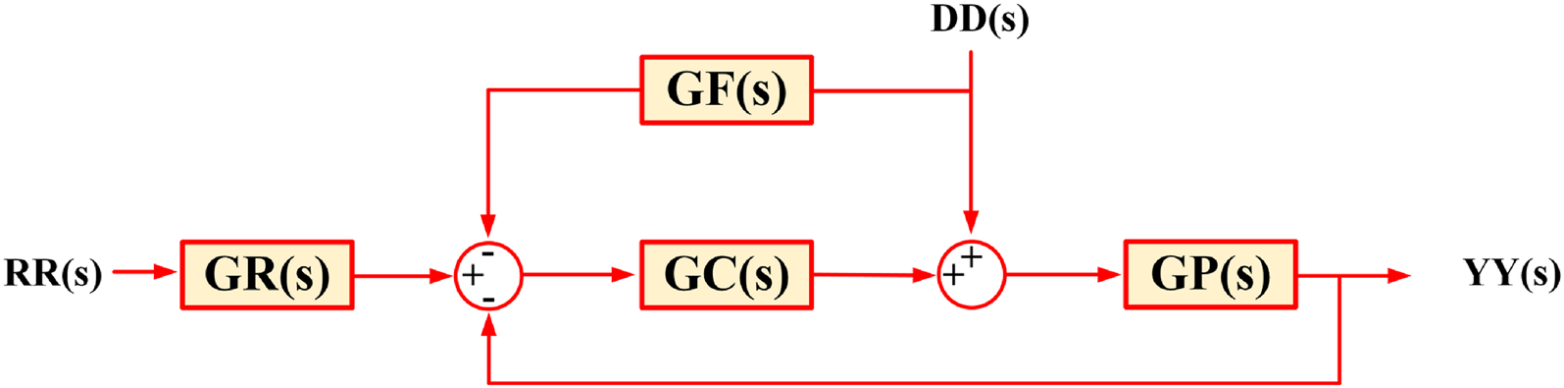
Block diagram of 3DOF controller.

A conventional controller has limitations as it only initiates corrective measures after a controlled variable deviates significantly from the reference value. A 3DOF controller has three closed-loop transfer functions, focusing on stability, response tailoring, and disturbance mitigation, allowing more freedom in controlling systems. Figure 13 shows the basic block diagram of the 3DOF controller.

In Fig. 13, *RR(s)*, *YY(s)*, and *DD(s)* represent the reference input, the output from the measuring device, and the external disturbance signal, respectively. *GR(s)*, *GC(s)*, and *GF(s)* represent the transfer functions of the reference input controller, the 1-DOF controller, and the feed-forward controller, respectively. *GP(s)* represents the transfer function of the plant. Within the proposed cascade controller framework, the output from the measuring device is computed as depicted in Fig. 13 and is provided in Eq. (31).

31 $$\begin{aligned} YY(s) = & GR\left( s \right)\frac{GC\left( s \right)GP\left( s \right)}{{1 + GC\left( s \right)GP\left( s \right)}}RR\left( s \right) \\ & + \frac{GP\left( s \right) - GP\left( s \right)GF\left( s \right)GC\left( s \right)}{{1 + GC\left( s \right)GP\left( s \right)}}DD\left( s \right) \\ \end{aligned}$$

**Figure 14:**
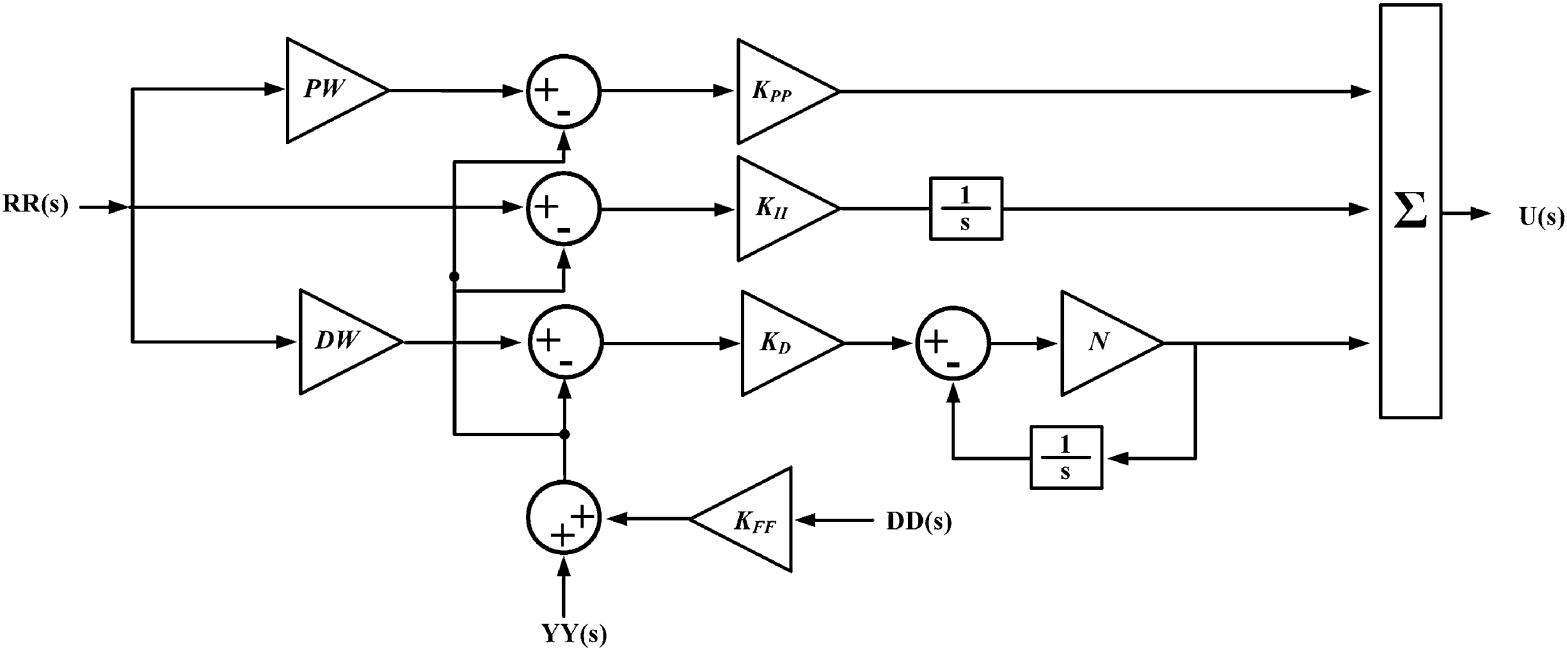
Basic structure of 3DOF-PID controller.

Figure 14 illustrates the fundamental structure of a 3DOF-PID controller. In this diagram, *PW* and *DW* represent the proportional and derivative weights, respectively. *N* is a derivative filter coefficient, which corresponds to the pole of the low-pass filter. *K*_*FF*_ signifies the feed-forward controller gain for the input *DD(s).* For the secondary controller, *K*_*PP*_, *K*_*I*_, and *K*_*DD*_ denote the proportional, integral, and derivative gains, respectively. The overall transfer function of the model, which is equivalent to the transfer function of the secondary controller (*Cs*(*s*)), is calculated considering one input at a time, as follows.

32 $$\begin{aligned} GR(s) = \frac{U\left( s \right)}{{YY\left( s \right)}} = & - \left[ {K_{PP} + \frac{{K_{II} }}{s} + K_{D} \left( {\frac{sN}{{s + N}}} \right)} \right] \\ = & - \left[ {\frac{{s^{2} \left( {NK_{D} + K_{PP} } \right) + s\left( {NK_{PP} + K_{II} } \right) + K_{II} N}}{{s\left( {S + N} \right)}}} \right] \\ \end{aligned}$$

33 $$\begin{aligned} GF(s) = \frac{U\left( s \right)}{{DD\left( s \right)}} = & - K_{FF} \left[ {K_{PP} + \frac{{K_{II} }}{s} + K_{D} \left( {\frac{sN}{{s + N}}} \right)} \right] \\ = & - K_{FF} \left[ {\frac{{s^{2} \left( {NK_{D} + K_{PP} } \right) + s\left( {NK_{PP} + K_{II} } \right) + K_{II} N}}{{s\left( {S + N} \right)}}} \right] \\ \end{aligned}$$

#### Structure 1PD-3DOF-PID controller

The proposed control system employs a secondary controller known as 3DOF-PID in conjunction with a primary controller, 1PD. In this configuration, the output of the primary controller serves as the set point input for the secondary controller. The inspiration for this novel controller stems from the synergistic integration of the strengths of both 1PD and 3DOF-PID controllers. The Cascade control scheme improves system performance in disturbances using multiple tuning loops, using metaheuristics-based design methodologies, and cascade control-based DoF in LFC.

However, despite its numerous advantages and remarkable attributes, the combination of 1PD and 3DOF-PID controllers, referred to as the 1PD-3DOF-PID controller, has received limited attention. Consequently, the authors are motivated to introduce this innovative and robust controller to the field of LFC research as a novel method to regulate system frequency with maximum effectiveness.

**Figure 15:**
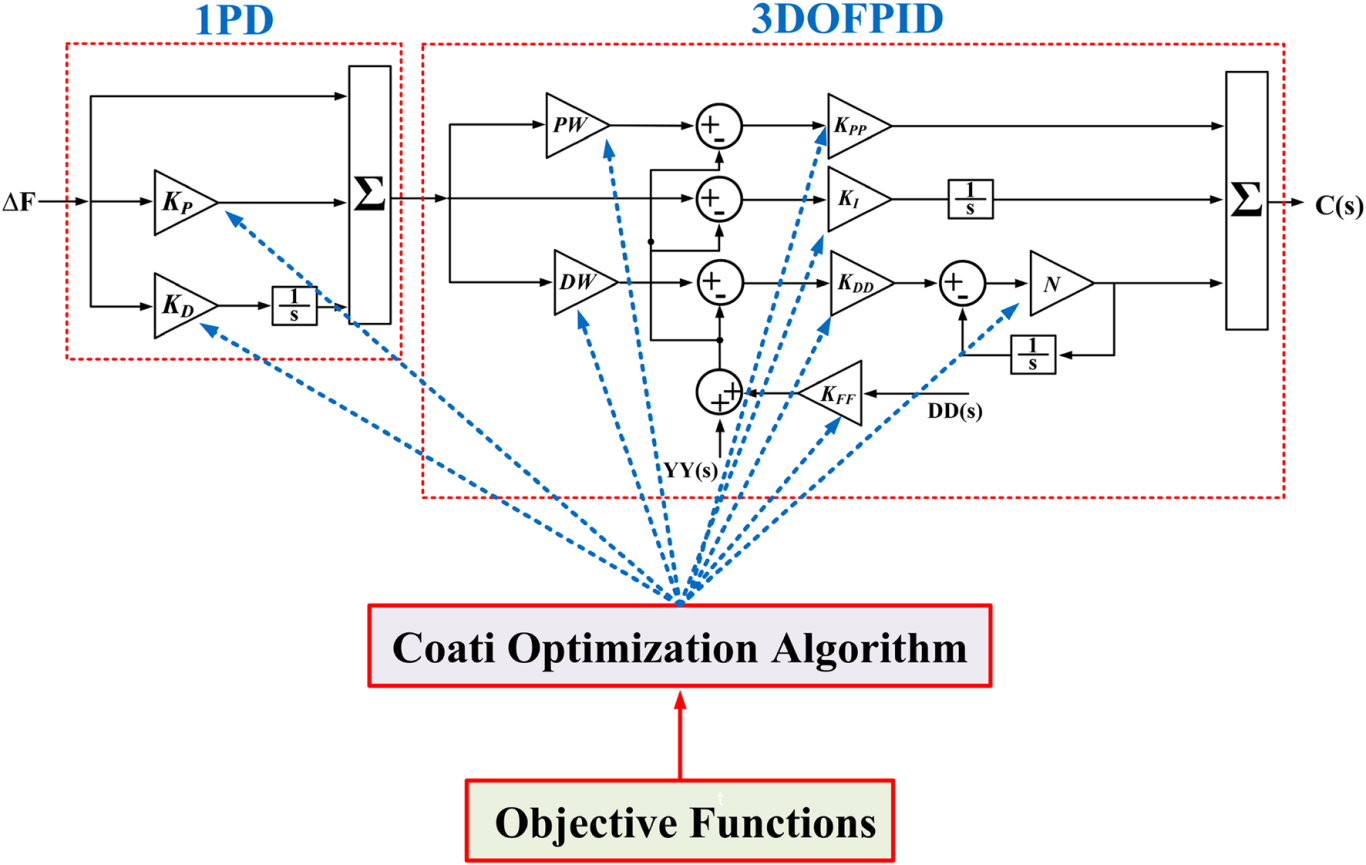
Structure of 1PD-3DOF-PID Controller.

The mathematical model of the 1PD-3DOF-PID controller is shown in Fig. 15.

## Implementation of the proposed LFC method: design and settings

This study employs three controllers, namely, 1PD-3DOF-PID, 3DOF-PID, and PID, with a primary focus on parameter adjustment to minimize objective functions. The 1PD-3DOF-PID controller serves as the main controller, subject to constraints defined by Eq. (34).

**Figure 16:**
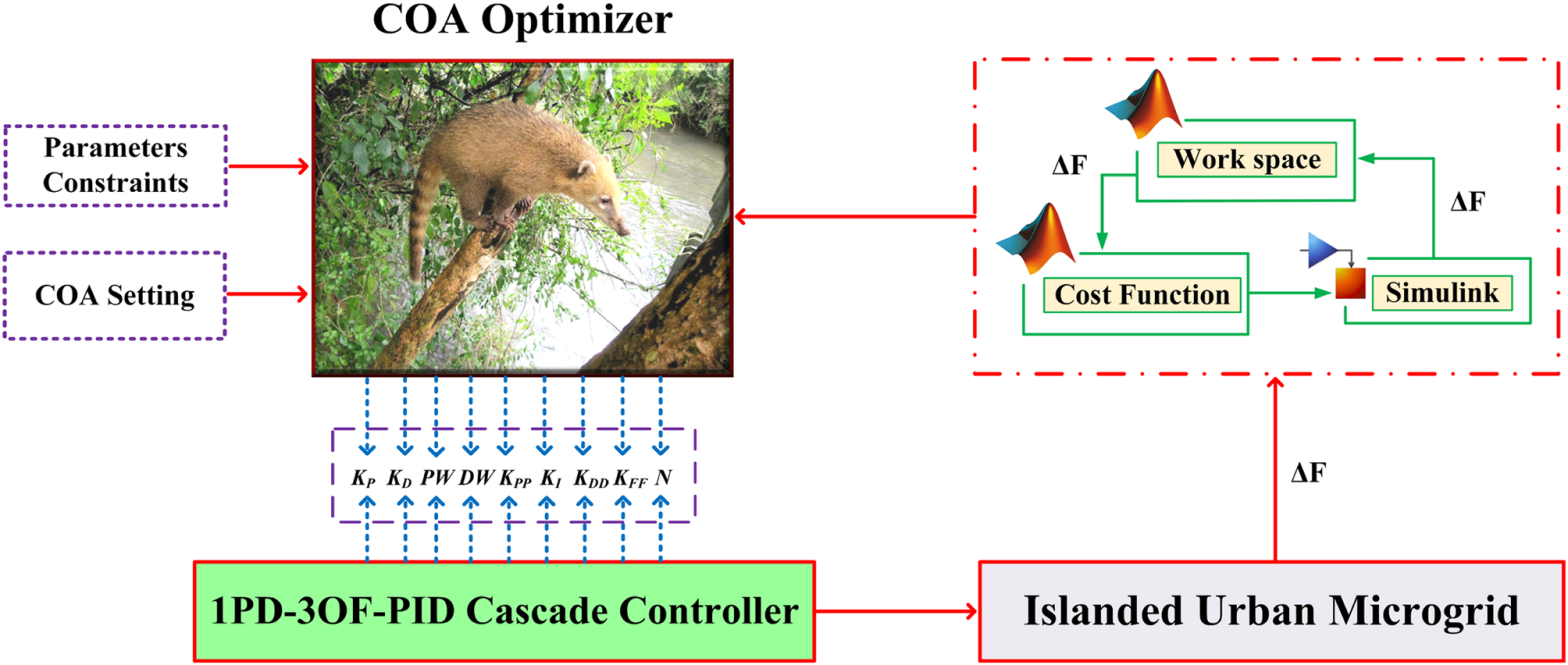
Flowchart of the control strategy.

Figure 16 displays a flowchart illustrating the control strategy and interaction between the Simulink MATLAB environment and workspace using frequency deviation parameters. Given the nature of the COA method as a population-based metaheuristic, it is essential to consider coatis as integral members within the algorithm’s population. Their positions within the search space fundamentally influence the determination of decision variable values. Estimating the appropriate search space can be achieved with precision by approximating the number of individuals or jackets involved.

In the design process, a pivotal factor that serves as a termination criterion is the number of iterations. Notably, the COA technique offers notable advantages, one of which is the absence of control parameters during its development. Consequently, there is no necessity for fine-tuning or adjustment of settings. In contrast, Table 3 provides a comprehensive overview of the control parameters harnessed by the RSA and WSO optimizers. Furthermore, some initial configurations, which are common to all three optimizers, are succinctly summarized in Table 4 below.

34 $$1PD - 3DOFPID:\left\{ {\begin{array}{*{20}c} {K_{P}^{\min } \le K_{P} \le K_{P}^{\max } } \\ {K_{D}^{\min } \le K_{D} \le K_{D}^{\max } } \\ {PW^{\min } \le PW \le PW^{\max } } \\ {DW^{\min } \le DW \le DW^{\max } } \\ {K_{PP}^{\min } \le K_{PP} \le K_{PP}^{\max } } \\ {K_{I}^{\min } \le K_{I} \le K_{I}^{\max } } \\ {K_{DD}^{\min } \le K_{DD} \le K_{DD}^{\max } } \\ {K_{FF}^{\min } \le K_{FF} \le K_{FF}^{\max } } \\ {N^{\min } \le N \le N^{\max } } \\ \end{array} } \right.$$

**Table 3:** Control parameters of RSA and WSO optimizers.

Algorithm	Parameter	Value
*WSO*	*f*_*min*_	0.07
	*f*_*max*_	0.75
	*τ*	4.126
	*a*_*0*_	6.25
	*a*_*1*_	100
	*a*_*2*_	0.0005
*RSA*	Sensitive parameters	*β* = 0.01
		*α* = 0.1
	Evolutionary Sense (ES)	ES: randomly decreasing values between − 2 and 2

**Table 4:** Initial settings common to all three optimizers of COA, RSA and WSO optimizers.

Initial settings		Value
Population size		25
Maximum number of iterations		30
Controller design time in MATLAB		20
Range of controller gains	*K*_*P*_	[0, 10]
	*K*_*D*_	[0, 10]
	*P*_*W*_	[0, 10]
	*D*_*W*_	[0, 10]
	*K*_*PP*_	[0, 10]
	*K*_*DD*_	[0, 10]
	*K*_*I*_	[0, 10]
	*K*_*FF*_	[0, 10]
	*N*	[40, 120]

## Simulated results

The proposed LFC strategy underwent validation using a MEVES-based IUMG, as illustrated in Fig. 9. The proposed methodology was rigorously evaluated through a series of case studies. The simulations were conducted using MathWorks MATLAB/Simulink software version R2023b (URL link: https://in.mathworks.com/products/simulink.html) on a personal computer equipped with a Microsoft Windows 11 operating system, an Intel® Core™ i5-14600K CPU (3.50 GHz), and 32 GB of RAM.

This research work incorporates meta-heuristic optimization approaches, namely COA, RSA, and WSO. The COA approach was selected as the primary optimizer for system design, while both RSA and WSO techniques were employed as evaluation optimizers. For the critical task of LFC, a combination of 1PD-3DOF-PID, 3DOF-PID, and PID controllers was employed. The effectiveness of the proposed control technique was demonstrated using real-time wind and solar data. Additionally, controller performance was rigorously assessed through time domain indices such as ISE, IAE, ITSE, and ITAE.

This evaluation framework employs four distinct scenarios to comprehensively assess the performance of the proposed IUMG system. These scenarios analyze the system’s capabilities across various key aspects:

1. **Efficiency (Scenarios I, II, and III):** This evaluation focuses on the system’s ability to handle load demand uncertainty. Three distinct scenarios (denoted as Scenarios I, II, and III) explore different load patterns to assess the system’s efficiency under varying load conditions.
2. **Robustness (Scenario IV):** Scenario IV delves into the system’s robustness by employing sensitivity analysis. This analysis specifically investigates how the system’s performance is affected by variations in its key parameters.
3. **Environmental and Sustainability Impacts:** A separate part is dedicated to analyzing the potential environmental and sustainability benefits associated with the proposed IUMG system.

It is crucial to emphasize that during the controller design process, the controller gains are initially computed through optimization methods for scenario I. Subsequently, this procedure is repeated for three additional scenarios to demonstrate the controllers’ performance.

### Evaluation of efficiency

To comprehensively assess the proposed controller’s performance under realistic operating conditions characterized by load demand uncertainty, the evaluation process employs three distinct scenarios. This selection of scenarios ensures a rigorous examination of the controller’s effectiveness across a varied range of operating conditions.

The evaluation of efficiency considers three distinct scenarios. This selection of scenarios ensures a comprehensive evaluation of the controller’s efficiency under various operating conditions.

#### Scenario I: Load pattern with a unit step

##### Controller parameter design

**Figure 17:**
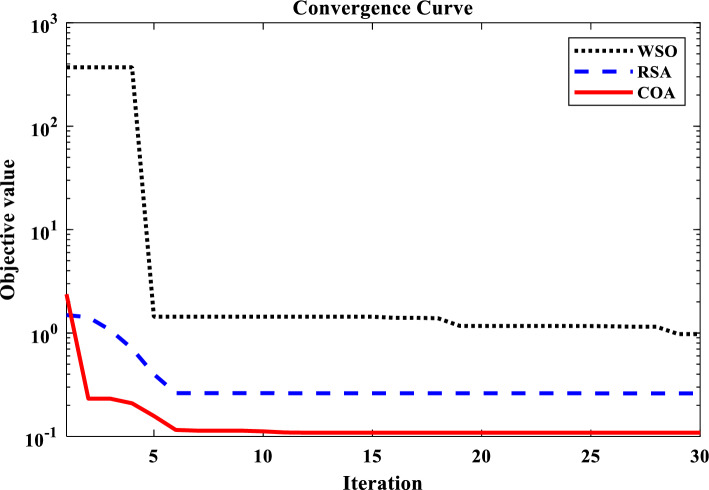
Convergence curve of optimization algorithms.

**Figure 18:**
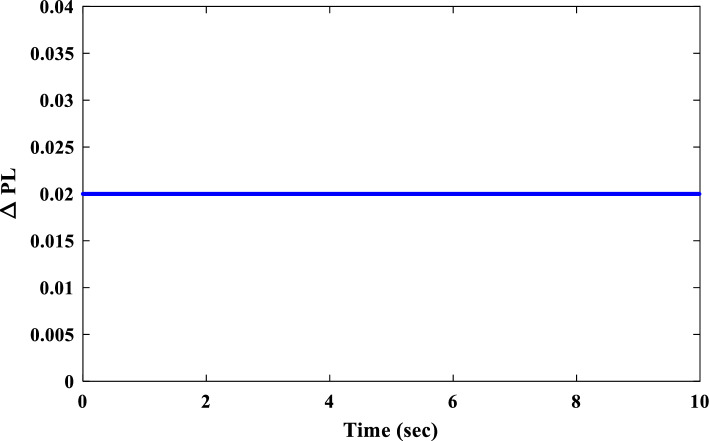
The pattern of step load changes in Scenario I.

Unit step change of 0.02 pu was applied to the IUMG load to facilitate the design of controller parameters for the first scenario. This step change served as the basis for optimizing the coefficients of three controllers: the proposed 1PD-3DOF-PID controller, a 3DOF-PID controller, and a PID controller. Three distinct optimization techniques (COA, WSO, and RSA) were employed for this purpose. Table 5 summarizes the resulting controller coefficients obtained through each optimization technique, adhering to the predefined parameter range constraints. Figure 17 depicts the convergence curves of these optimization methods, visually demonstrating their effectiveness in addressing the LFC challenges within the IUMG optimization framework. The specific pattern of step load changes employed in scenario I is illustrated in Fig. 18.

**Table 5:** Optimized gains of controllers in IUMG.

Gains	1PD-3DOF-PID			3DOF-PID			PID		
	WSO	RSA	COA	WSO	RSA	COA	WSO	RSA	COA
KP	1.184	5.000	0.340	–	–	–	–	–	–
KD	0.648	4.752	1.145	–	–	–	–	–	–
PW	2.856	0.014	3.857	8.151	6.748	2.125	–	–	–
DW	3.105	0.145	6.145	9.145	2.125	2.001	–	–	–
KPP	6.001	5.257	3.258	2.582	0.111	9.950	4.585	0.589	7.859
KI	4.585	2.014	7.077	1.258	0.963	0.148	6.258	1.001	7.058
KDD	7.859	4.189	3.145	2.785	0.116	9.285	0.015	5.350	9.856
KFF	1.585	2.589	7.558	–	–	–	–	–	–
N	75	110	115	45	40	88	72	85	110

##### Frequency response analysis of scenario I

**Figure 19:**
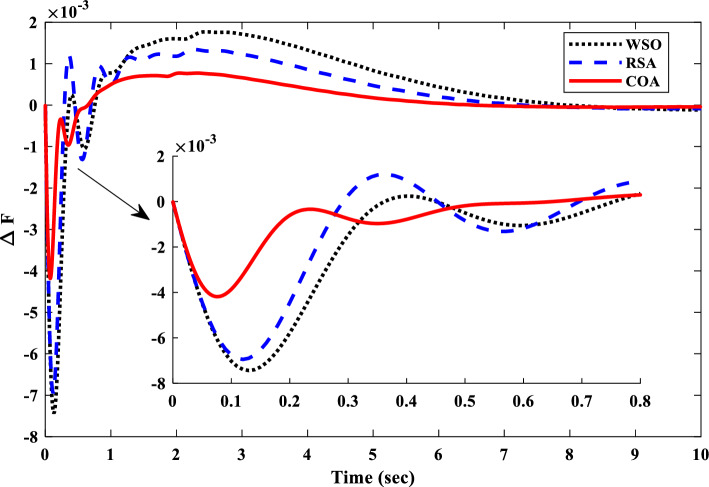
IUMG frequency response for 1PD-3DOF-PID controller under different algorithms – Scenario I.

Figure 19 presents the IUMG frequency response achieved by the 1PD-3DOF-PID controller under each optimization algorithm within Scenario I. The results highlight the superiority of the COA-based design, exhibiting faster response and improved performance indices compared to the other controllers. This observation underscores the robustness of the COA technique in controller design for IUMG frequency regulation.

**Figure 20:**
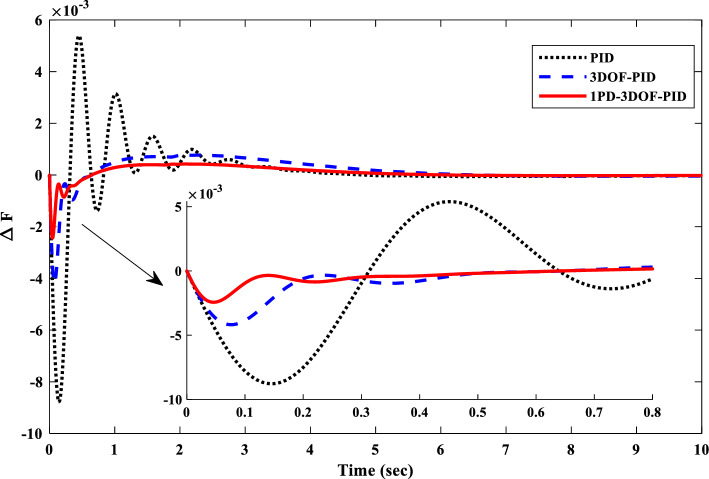
IUMG frequency response of various controllers based on COA– Scenario I.

Figure 20 further emphasizes the efficacy of the 1PD-3DOF-PID controller designed using the COA technique under varying step load conditions in Scenario I. As evident from the figure, the proposed controller demonstrates exceptional performance in regulating IUMG load fluctuations. The inherent domain structure of the controller facilitates a faster settling time and reduced frequency deviations.

##### Power sources response analysis of scenario I

**Figure 21:**
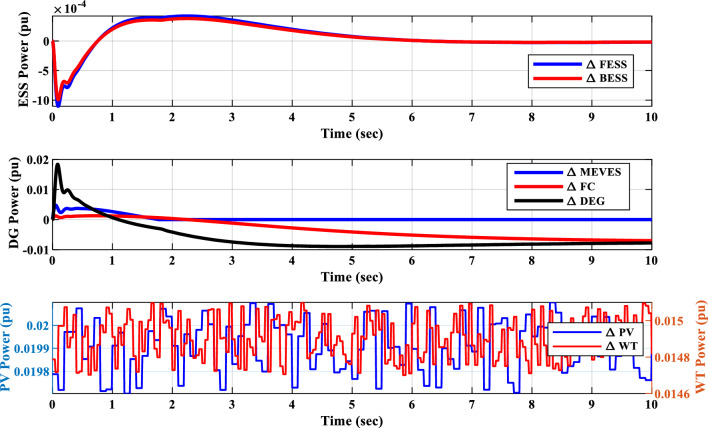
Changes in the output power of DER units– Scenario I.

Figure 21 portrays the variations in the output power of different sources within the IUMG during scenario I. It distinguishes between uncontrollable sources (WTG and PV systems) with fluctuating power output and controllable sources (DEG, FC, and MEVES) whose output is adjusted based on load and frequency deviations. The figure also depicts the participation rate of each controllable source during load changes, with the DEG unit exhibiting a higher involvement due to its faster dynamics. Also, Fig. 21 illustrates the output power of the BESS and FESS. The fluctuations in power output from these storage devices reflect their charge and discharge patterns in response to IUMG frequency variations, ultimately maintaining power balance within the microgrid.

##### Comparative evaluation of scenario I

Table 6 presents a comprehensive comparison of the controllers’ performance metrics, including settling time, overshoot, undershoot, and the numerical values of evaluation indices (ISE, ITSE, IAE, and ITAE). This data analysis reveals the clear superiority of the proposed 1PD-3DOF-PID controller. Notably, it achieves a settling time 3.93 s faster than the alternative controllers. Additionally, the evaluation indices for the proposed controller (0.0001075 and 0.40062) are significantly lower compared to the others, further solidifying its exceptional performance.

**Table 6:** Time domain evaluation indexes – scenario I.

Controller	PID	3DOF-PID	1PD-3DOF-PID
Undershoot	0.0087724	0.0041848	0.0024386
Overshoot	0.0053909	0.00077271	0.00042568
Settling time	6.0296	5.5524	3.9359
ISE	0.0019602	0.00058697	0.00021034
ITSE	0.00081355	0.00039694	0.0001075
IAE	0.54367	0.41444	0.24519
ITAE	0.66133	0.80214	0.40062

#### Scenario II: Load pattern with random steps

**Figure 22:**
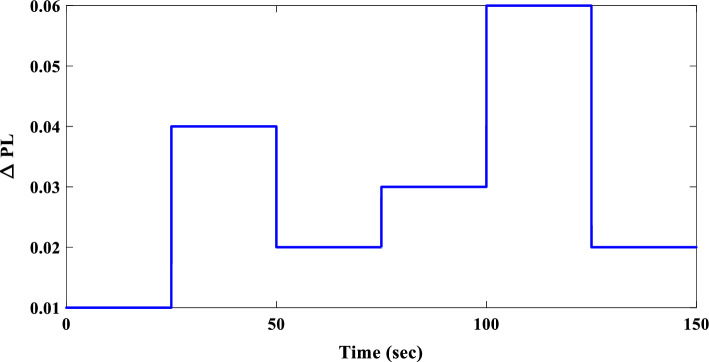
Pattern of random step load changes—Second II.

To evaluate the proposed controller’s robustness under realistic operating conditions, time-varying load profiles were implemented within the IUMG. These profiles, depicted in Fig. 22, simulate load fluctuations with minimum and maximum values of 0.01 and 0.04 pu, respectively. The objective is to assess the controller’s efficacy in maintaining frequency regulation and system stability amidst dynamic load variations. It is noteworthy that the controller coefficients, optimized for scenario I, are retained for this scenario.

##### Frequency response analysis of scenario I

**Figure 23:**
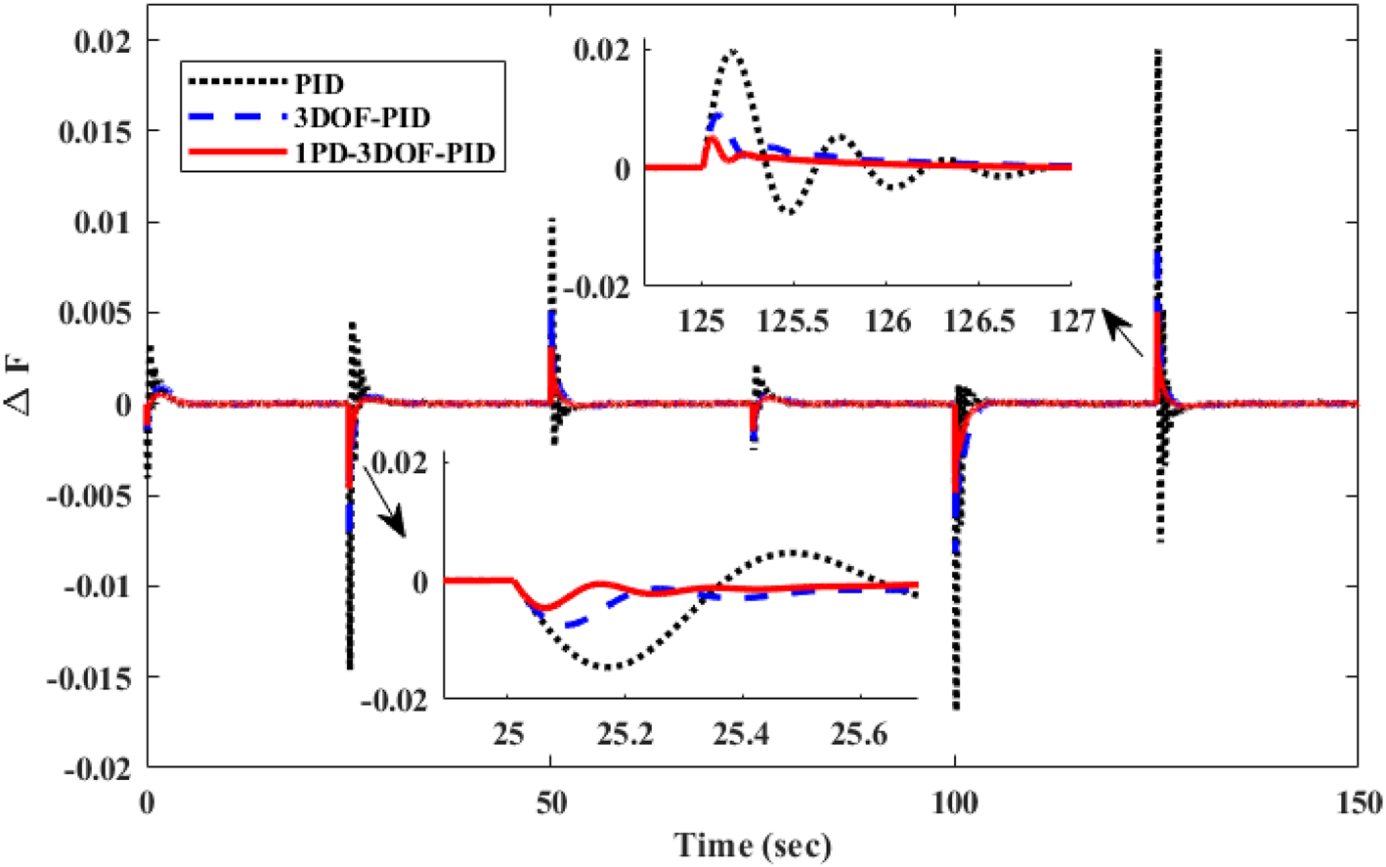
IUMG frequency response of various controllers based on COA – Scenario II.

Figure 23 compares the IUMG frequency response under various random step load disturbances for the 1PD-3DOF-PID, 3DOF-PID, and PID controllers, all optimized using the COA technique. The results demonstrate the superior performance of the 1PD-3DOF-PID controller in regulating these load changes, achieving faster damping of frequency deviations.

**Figure 24:**
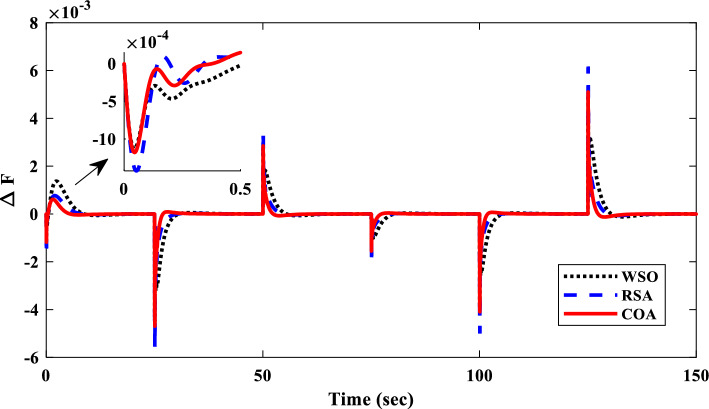
IUMG frequency response for 1PD-3DOF-PID controller under different algorithms – Scenario II.

Figure 24 further reinforces this observation. It presents the microgrid’s frequency response under variable step load disturbances when employing the 1PD-3DOF-PID controller optimized through the three different algorithms (COA, WSO, RSA). The data confirms that the COA-optimized 1PD-3DOF-PID controller effectively manages variable load changes and efficiently mitigates frequency deviations.

##### Power sources response analysis of scenario II

**Figure 25:**
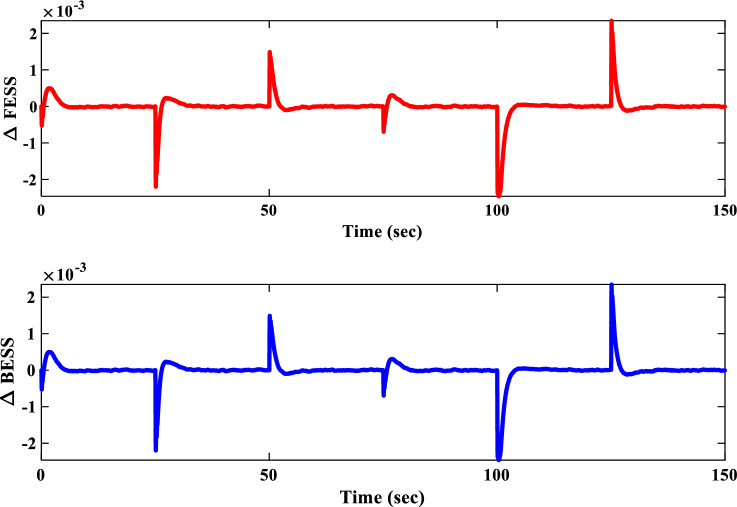
Changes in the output power of FESS and FESS units – Scenario II.

**Figure 26:**
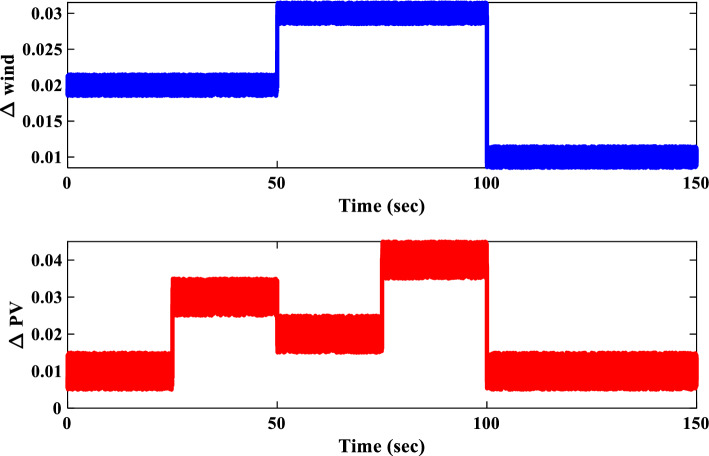
Changes in the output power of PV and WTG unit – Scenario II.

**Figure 27:**
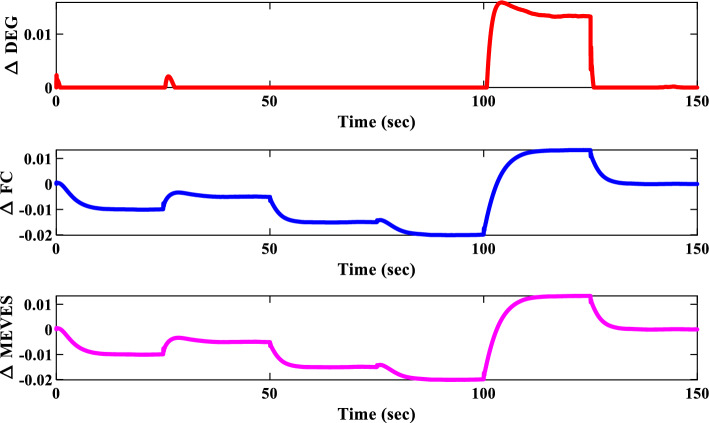
Changes in the output power of DEG, FC and MEVES units – Scenario II.

Figures 25, 26 and 27 illustrate the output power variations of various sources within the IUMG during scenario II. The power contribution of each controllable source (FESS, BESS, MEVES, FC, DEG) approximately mirrors the observations from Scenario I. Figure 25 depicts the power contributions of the FESS and BESS units. The fluctuations in these storage devices reflect their charging and discharging patterns in response to the combined effects of load variations and power balance limitations. This scenario simulates a situation where renewable energy sources exhibit unpredictable and time-varying power output. Figure 26 shows the changes in the output power of the PV and WTG units, highlighting the difference in RES output compared to Scenario I.

##### Comparative evaluation of scenario II

Table 7 provides a comprehensive comparison of key time-domain indices for various algorithms and the three controllers. These indices include settling time, overshoot, undershoot, and the numerical values of evaluation indices (ITAE, ITSE). This comparison facilitates the identification of the most effective controller and optimization algorithm. Notably, Table 7 reinforces the superior performance of the 1PD-3DOF-PID controller, evidenced by its lower ITAE (115.5203) and ITSE (0.23262) values.

**Table 7:** Time domain evaluation indexes – scenario II.

Controller	PID	3DOF-PID	1PD-3DOF-PID
Undershoot	0.015214	0.0076936	0.0046924
Overshoot	0.019475	0.0088742	0.0051008
Settling time	13.04134	12.99324	12.79359
ISE	0.021047	0.0090314	0.0030456
ITSE	1.7585	0.7034	0.23262
IAE	3.3649	2.9368	1.6985
ITAE	240.8961	200.1107	115.5203

#### Scenario III: Load pattern with pulsed fluctuations

**Figure 28:**
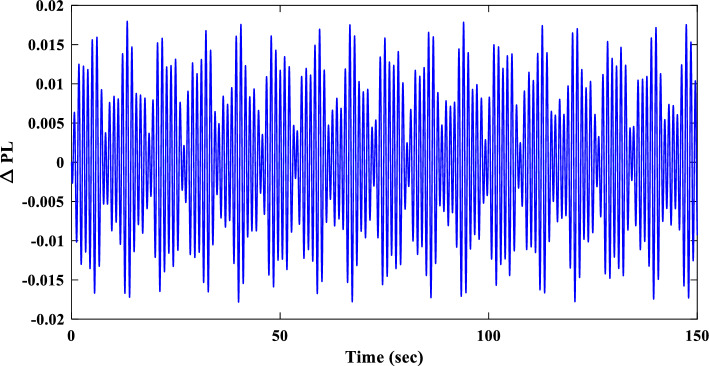
Load pattern with fluctuations pulsed – Scenario III.

Scenario III investigates the controller’s resilience under dynamic load variations using a pulsed load change pattern. This time-dependent model, depicted in Fig. 28, replicates load uncertainty with fluctuations reaching a maximum value of 0.018 pu at a frequency of 50 Hz. The objective is to assess the controller’s performance in mitigating these challenging conditions.

##### Frequency response analysis of scenario III

**Figure 29:**
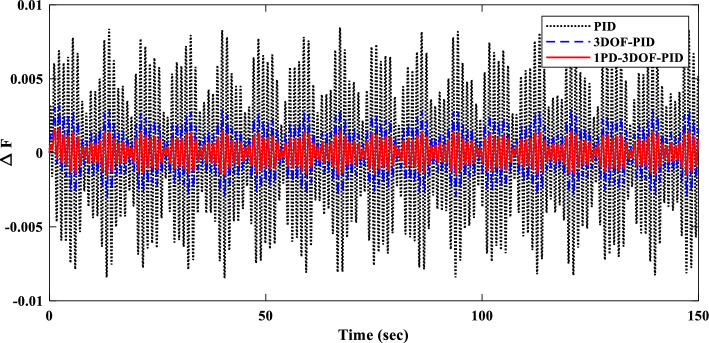
IUMG frequency response of various controllers based on COA—Scenario III.

Figure 29 compares the frequency response of the 1PD-3DOF-PID controller optimized using the COA technique with the 3DOF-PID and PID controllers under pulsed load fluctuations in scenario III. This comparison reinforces the effectiveness of the COA technique in designing a robust controller, even under uncertain circumstances. The figure confirms the successful performance of the 1PD-3DOF-PID controller in managing load fluctuations while minimizing frequency deviations.

##### Power sources response analysis of scenario III

**Figure 30:**
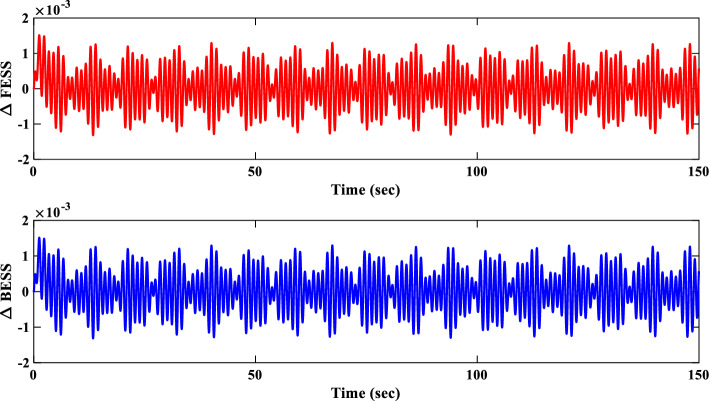
Changes in the output power of FESS and FESS units – Scenario III.

**Figure 31:**
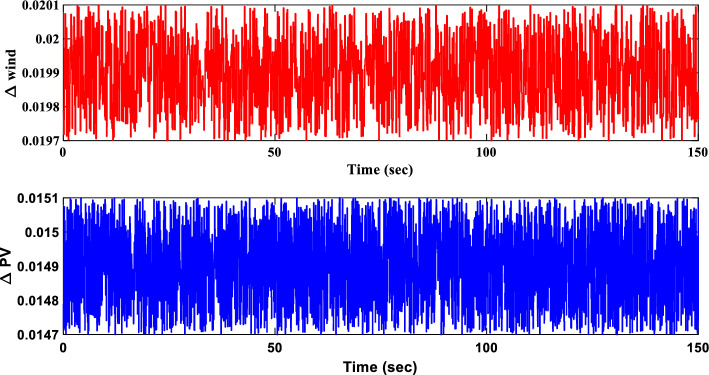
Changes in the output power of PV and WTG unit – Scenario III.

**Figure 32:**
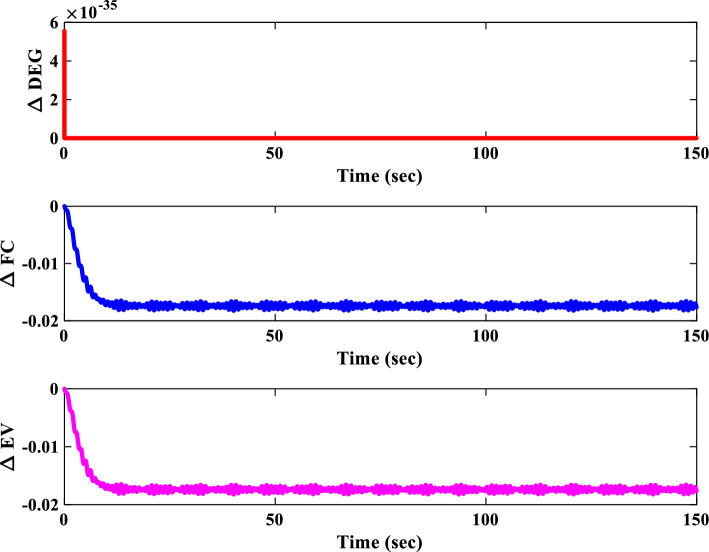
Changes in the output power of DEG, FC and MEVES units – Scenario III.

Figures 30, 31 and 32 illustrate the output power variations of various IUMG sources during scenario III. Figure 30 depicts the fluctuating load profile within the microgrid, alongside the corresponding charging and discharging patterns of the BESS and FESS units. These variations highlight the system’s efforts to maintain power balance amidst dynamic load changes. Figure 31 showcases the inherent variability in power generation from uncontrollable renewable sources (WTG and PV) due to factors like wind and solar conditions. These unpredictable fluctuations, along with temporal shifts, introduce random alterations to the system, enhancing the evaluation’s realism.

##### Comparative evaluation of scenario II

Table 8 presents a comprehensive comparison of various time-domain indicators derived from the simulations. Notably, the ITSE and ITAE indices consistently show lower values for the 1PD-3DOF-PID controller compared to other controllers across all scenarios. This observation underscores the controller’s superior performance throughout the evaluation process.

**Table 8:** Time domain evaluation indexes – scenario III.

Controller	PID	3DOF-PID	1PD-3DOF-PID
Undershoot	0.031948	0.01577	0.0094977
Overshoot	0.029853	0.014644	0.0088833
Settling time	12.79336	12.97733	12.94326
ISE	0.11414	0.049536	0.018482
ITSE	7.2644	3.2961	1.2332
IAE	8.3611	7.5381	4.625
ITAE	537.7544	507.2915	307.635

### Robustness evaluation: sensitivity analysis of system parameter changes

This subsection investigates the robustness of the 1PD-3DOF-PID controller in the presence of parametric uncertainties arising from modeling variations. A sensitivity analysis is conducted through Scenario IV, which involves step changes in key IUMG parameters. Specifically, the analysis examines the effects of modifying the following parameters by 25% from their nominal values: Inertia Constant (*M*), Load Damping Coefficient (*D*), Fuel Cell Time Constant (*T*_*FC*_), MEVES Time Constant (*T*_*MEVES*_), Governor Time Constant (*T*_*G*_).

#### Uniaxial sensitivity analysis

The IUMG is first evaluated under settings identical to Scenario I. For each individual parameter change, all other unit settings remain consistent with Scenario I. This allows for a detailed analysis of the resulting time-domain evaluation indices, presented in Table 9. The table explores the impact of each parameter uncertainty on the system’s performance in single execution mode. As evident from the table, the deviations in time-domain indices from their nominal values are relatively minor.

**Table 9:** Time domain evaluation indexes – scenario IV.

Parameter	Range changes (%)	Uundershoot	Settling time	Overshoot	ITSE	ITAE
M	− 25	0.0027114	5.4569	0.00042589	0.00010698	0.34349
	+ 25	0.0022472	5.6327	0.00042727	0.00010785	0.34506
D	− 25	0.0024394	5.5701	0.00042563	0.00010661	0.34361
	+ 25	0.0024378	5.5731	0.00042618	0.00010682	0.34413
*T*_*FC*_	− 25	0.0024002	5.3737	0.00041210	0.00096602	0.32851
	+ 25	0.0024645	5.6735	0.00043483	0.00011574	0.36473
*T*_*MEVES*_	− 25	0.0024386	5.5719	0.00042568	0.00010620	0.34319
	+ 25	0.0024386	5. 5719	0.00042568	0.00010620	0.34319
*T*_*G*_	− 25	0.0024386	5. 5719	0.00042568	0.00010620	0.34319
	+ 25	0.0024002	5.3737	0.00041210	0.00099602	0.31851

#### Multiaxial sensitivity analysis

**Figure 33:**
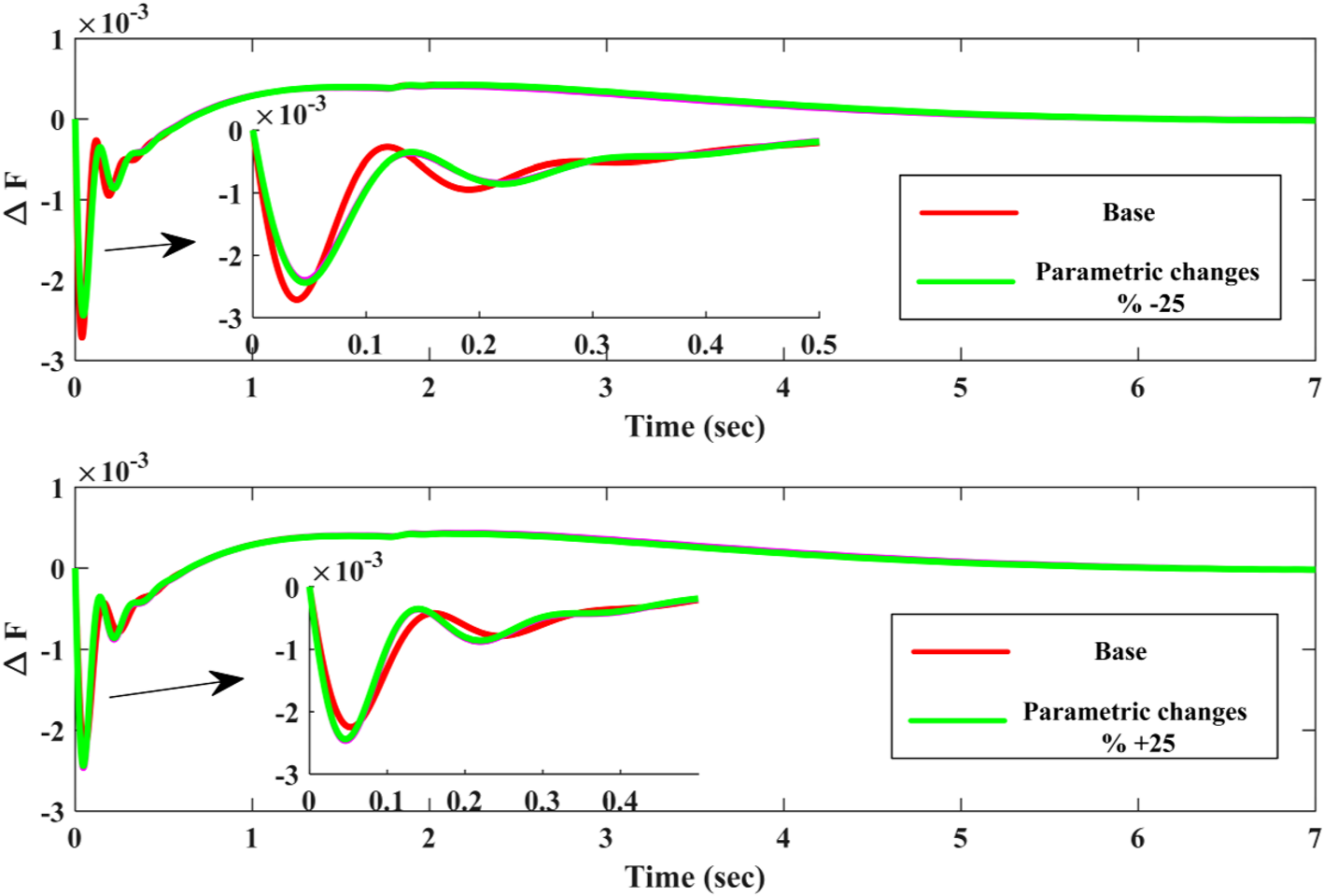
IUMG frequency response for 1PD-3DOF-PID controller under COA – Scenario IV.

To comprehensively assess the IUMG’s sensitivity to a wider range of parameter uncertainties, a multiaxial sensitivity analysis is conducted. In this analysis, all previously identified parameter uncertainties are applied simultaneously to the IUMG model. All other settings are maintained in accordance with Scenario I, facilitating a direct comparison with the baseline case (Scenario I). Figure 33 depicts the IUMG’s frequency response for the 1PD-3DOF-PID controller designed using the COA technique under Scenario IV. This scenario incorporates all previously identified parameter uncertainties, allowing for an assessment of the controller’s performance under these combined variations. The figure also includes a comparison with the frequency response obtained in Scenario I (baseline case). This comparison allows for a direct evaluation of the controller’s robustness in mitigating frequency deviations under more comprehensive parameter uncertainties.

The combined findings from Table 9 and Fig. 33 demonstrate the robustness of the proposed control scheme under variations in IUMG parameters, ensuring system stability. The sensitivity analysis highlights that once the controller settings are established, they likely do not require significant adjustments for future parameter changes within the IUMG.

### Evaluating potential environmental and sustainability impacts

The proposed control method, the 1PD-3DOF-PID controller designed using the OA technique (as 1PD-3DOF-PID/COA), offers potential benefits for the environmental and sustainability performance of IUMG systems.

1. **Reduced emissions and improved fuel economy:** The 1PD-3DOF-PID/COA controller demonstrably enhances the dynamic performance of the IUMG by effectively mitigating frequency deviations and load fluctuations. This improved system stability directly translates to potential reductions in greenhouse gas emissions. By ensuring optimal operation of controllable power sources (e.g., Diesel Engine Generators) and minimizing unnecessary load shedding events, the controller promotes a more efficient use of fossil fuels, leading to lower overall emissions.
2. **Enhanced integration of RES:** The robust performance of the 1PD-3DOF-PID/COA controller under varying operating conditions facilitates a smoother integration of renewable energy sources with inherent variability (e.g., WTG and PV systems). The controller’s ability to effectively manage these fluctuations in power generation allows for a greater reliance on renewable resources, ultimately contributing to a more sustainable energy mix for the IUMG.
3. **Improved system efficiency and reduced losses:** The superior performance of the 1PD-3DOF-PID/COA controller in regulating system frequency and power flow potentially translates to reduced energy losses within the IUMG. By minimizing unnecessary power fluctuations and maintaining stable operating conditions, the controller promotes more efficient energy transmission and distribution. This can lead to overall energy savings and a reduction in the IUMG’s environmental footprint.
4. **Limitations and considerations:** While the 1PD-3DOF-PID/COA controller offers promising environmental and sustainability benefits, it is essential to acknowledge certain limitations. The actual reduction in emissions and improvement in fuel economy achieved will depend on the specific configuration of the IUMG and the energy mix utilized. Additionally, further research might be necessary to quantify the exact impact of the controller on energy losses within the system.

In general, the 1PD-3DOF-PID/COA controller presents a promising approach for enhancing the environmental and sustainability performance of IUMGs. By promoting improved system stability, efficient operation of power sources, and a greater reliance on renewable energy, this controller contributes to a more sustainable and environmentally friendly microgrid operation. Future research efforts directed at quantifying the specific environmental benefits and potential trade-offs associated with this control strategy are recommended.

## Discussion and analysis of results

This section delves into a detailed analysis and discussion of the time-domain evaluation results obtained from the various scenarios. The primary focus lies on comparing the performance of the three controllera (PID, 3DOF-PID, and 1PD-3DOF-PID) across Scenarios I-III using the metrics of undershoot, overshoot, and settling time. Subsequently, the analysis shifts to assess the impact of parameter uncertainties in Scenario IV on these same performance indicators.

### Scenarios I–III: Comparison of controllers performance

Table 10 summarizes the time-domain evaluation indices for Scenarios I–III, allowing for a direct comparison of the three PID controllers:

1. **Undershoot:** Across all three scenarios, the 1PD-3DOF-PID controller consistently exhibits the lowest undershoot values, followed by the 3DOF-PID and PID controllers, respectively. This trend suggests that the 1PD-3DOF-PID controller demonstrates superior ability to minimize voltage sags during transient events.
2. **Overshoot:** Similar to undershoot, the 1PD-3DOF-PID controller exhibits the lowest overshoot values in all scenarios. This indicates its effectiveness in mitigating voltage spikes following disturbances.
3. **Settling time:** The 1PD-3DOF-PID controller generally achieves the fastest settling time in Scenarios I and II, signifying its quicker response in stabilizing system frequency after transients. While the settling time differences between the controllers become less pronounced in Scenario III, the 1PD-3DOF-PID controller still maintains a slight advantage.

**Table 10:** Time domain evaluation indexes _ Scenarios I–III.

Scenarios	Controller	PID	3DOF-PID	1PD-3DOF-PID
I	Undershoot	0.0087724	0.0041848	0.0024386
	Overshoot	0.0053909	0.00077271	0.00042568
	Settling time	6.0296	5.5524	3.9359
	ISE	0.0019602	0.00058697	0.00021034
	ITSE	0.00081355	0.00039694	0.0001075
	IAE	0.54367	0.41444	0.24519
	ITAE	0.66133	0.80214	0.40062
III	Undershoot	0.015214	0.0076936	0.0046924
	Overshoot	0.019475	0.0088742	0.0051008
	Settling time	13.04134	12.99324	12.79359
	ISE	0.021047	0.0090314	0.0030456
	ITSE	1.7585	0.7034	0.23262
	IAE	3.3649	2.9368	1.6985
	ITAE	240.8961	200.1107	115.5203
III	Undershoot	0.031948	0.01577	0.0094977
	Overshoot	0.029853	0.014644	0.0088833
	Settling time	12.79336	12.97733	12.94326
	ISE	0.11414	0.049536	0.018482
	ITSE	7.2644	3.2961	1.2332
	IAE	8.3611	7.5381	4.625
	ITAE	537.7544	507.2915	307.635

### Scenario IV: Impact of parameter uncertainties

Scenario IV focuses on the robustness of the control scheme by introducing parameter uncertainties. Table 9 provides the time-domain evaluation indices for this scenario:

1. **Undershoot:** The variations in undershoot values under parameter uncertainties are relatively minimal, ranging from 0.0022472 to 0.0027114.
2. **Overshoot:** Similar to undershoot, the changes in overshoot values are negligible, remaining within a narrow range of 0.00041210 to 0.00043483.
3. **Settling time:** The settling time exhibits a slightly wider range of variation (5.3737 to 5.6735) compared to undershoot and overshoot. However, these variations are still relatively small, suggesting that the 1PD-3DOF-PID controller maintains a robust performance even under parameter uncertainties.

The analysis of time-domain performance highlights the effectiveness of the 1PD-3DOF-PID controller. It consistently achieves superior performance in terms of undershoot, overshoot, and settling time compared to the other PID controllers across Scenarios I–III. Additionally, Scenario IV demonstrates the robustness of the 1PD-3DOF-PID controller, as its performance remains relatively stable even with parameter uncertainties.

## Advantages of the proposed approach

The The primary objective of this paper is to introduce a new LFC structure for the development of a novel and robust controller, i.e, 1PD-3DOF-PID, within an IUMG, leveraging a bio-inspired algorithm known as the COA. Several key considerations have been integral to the development of this proposed strategy, each of which holds significant importance in the eventual implementation of the strategy:

1. **Utilization of COA technique:** This state-of-the-art metaheuristic algorithm has been employed in the proposed control strategy. Its ease of design and adaptability to various controllers make it a promising choice.
2. **Flexible LFC strategy:** The proposed LFC strategy, incorporating recommended mobile EV energy storage, is designed to accommodate diverse microgrid configurations. These configurations may encompass distinct combinations of loads, renewable energy sources, and grid topologies.
3. **Computational efficiency:** The advocated cascade control technique is noteworthy for its computational efficiency. This characteristic is pivotal for both control system performance and practical implementation.
4. **Simulation and evaluation:** In the final phase, extensive simulations were conducted using various operating parameters relevant to the IUMD. These simulations served to assess the effectiveness and robustness of the proposed framework. The insights garnered from these evaluations determined the success of the unique control mechanism described herein.

## Conclusion and remark

This paper presented a novel approach to LFC analysis within IUMGs by introducing a COA Technique-optimized 1PD-3DOF-PID controller. The COA technique, inspired by the behavior of coatis, offers a unique optimization methodology with several advantages. Its parameter-free nature eliminates the need for complex control parameter tuning, and its effective balance between exploration and exploitation facilitates rapid convergence towards optimal solutions, particularly valuable for challenging optimization problems in IUMGs. The proposed control strategy’s performance was evaluated against various scenarios within the IUMG. Comparisons were made with alternative controllers, including the 1PD-3DOF-PID-based COA algorithm, RSA, and WSO algorithm. Additionally, established control techniques like 3DOF-PID and PID were included in the evaluation, confirming the effectiveness of the proposed approach. Notably, the 1PD-3DOF-PID controller with COA demonstrated significantly improved response times, effectively mitigating undershoots, overshoots, and settling time across diverse operating conditions. Simulation results showcased enhanced frequency responses within the IUMG, further validating the positive impact of the proposed control strategy. Moreover, robustness analysis subjected the COA Technique-optimized 1PD-3DOF-PID controller to ± 25% variations in key parameters, confirming its resilience to parameter uncertainties.

## Suggestions for future research

Building upon the promising results achieved with the COA Technique-optimized 1PD-3DOF-PID controller, future research efforts could explore several exciting avenues:

1. **Application to complex IUMG configurations:** Expanding the control strategy’s application to more intricate IUMG configurations that integrate diverse RES and advanced energy storage solutions would offer valuable insights into its scalability and adaptability.
2. **Exploration of 1PD-3DOF-PID design and optimization:** Continued investigation into the design and optimization methodologies for the 1PD-3DOF-PID controller presents opportunities for further performance improvements. This could involve exploring new meta-heuristic approaches for controller gain optimization.
3. **Integration of additional units and controllers:** Future research could delve into incorporating additional units and controllers within the secondary frequency control loop of IUMGs, potentially requiring detailed modeling enhancements for each energy source.
4. **Hybrid control strategies and higher DoF controllers:** Investigating the potential of employing hybrid control strategies and exploring more complex controllers with higher DoFcould be fruitful avenues for enhancing system responsiveness, although these approaches may present significant design challenges.

## Data Availability

The datasets used and/or analysed during the current study available from the corresponding author on reasonable request.

## Abbreviations

MGs: Microdrids
LFC: Load frequency control
IUMG: Islanded urban microgrid
COA: Coati optimization algorithm
RSA: Reptile search algorithm
WSO: White Shark optimizer
1PD-3DOF-PID: 1 + proportional (P) + derivative(D) -three degree of freedom (3DOF) proportional (P)-integral (I)-derivative (D) (PID)
ISE: Integral of squared error
IAE: Integral of absolute error
ITSE: Integral of time multiplied by squared error
ITAE: Integral of time multiplied by absolute error
RESs: Renewable energy sources
DEG: Diesel engine generator
WTG: Wind turbine generator
PV: Photovoltaic
ESSs: Energy storage systems
FCs: Fuel cells
DG: Distributed generation
BESS: Battery energy storage system
FESS: Flywheel energy storage system
pu: Perunit
EV: Electric vehicle
MEVES: Mobile EV energy storage
DERs: Distributed energy resources
PI: Proportional integral
DoF: Degrees of freedom
2DoF: Two-degree of freedom
3DoF: Three-degree of freedom
PID: Proportional integral derivative
FO: Fractional order
V2G: Vehicle-to-grid
SOS: State of charge
TEM: Total energy model
t_sim_: Simulation time
CB: Circuit breaker

## List of symbols

*P*_*wind*_: WTG output power
*P*_*mech*_: Mechanical power
*η*: WTG efficiency
*ρ*: WTG air density
*λ*: Tip speed ratio
*β*: Blade pitch angle
*ω*_*r*_: Angular velocity of the rotor
*R*: Blade length
*V*: Wind speed
*C*_*P*_: Power capturing efficiency
*ΔP*_*WTG*_: Changes of WTG output power
*G*_*WTG*_*(s)*: Linearized first-order transfer function of WTG
*K*_*WTG*_: WTG gain
*T*_*WTG*_: WTG time constant
*P*_*PV*_: PV output power
*φ*: Conversion efficiency of the PV array
*S*: Effective region of photovoltaic panels
*ξ*: Sign of solar irradiance
*T*_*A*_: Ambient temperature (standard operational value in 25 °C)
*ΔP*_*PV*_: Changes of PV output power
*G*_*PV*_*(s)*: Linearized first-order transfer function of PV
*K*_*PV*_: PV gain
*T*_*PV*_: PV time constant
*G*_*BESS*_*(s)*: Linearized first-order transfer function of BESS
*G*_*FESS*_*(s)*: Linearized first-order transfer function of FESS
*ΔP*_*BESS*_: Changes of BESS output power
*ΔP*_*F*__*ESS*_: Changes of FESS output power
*T*_*BESS*_: BESS time constant
*T*_*FESS*_: FESS time constant
*ΔP*_*MEVES*_: Changes of MEVES output power
*E*_*control*_: Total energy of all the controllable MEVES
*N*_*control*_: Total number of controllable MEVES
*N*_*Initial*_: Initial number of controllable MEVES
*N*_*control_in*_: Number of MEVES transferring from charging state to controllable state
*N*_*plug_out*_: Number of MEVES transferring from controllable state to driving state
*C*^***^_*kW*_: Inverter capacity of the MEVES energy storage unit
*C*^***^_*kWh*_: Battery capacity of the MEVES energy storage unit
*G*_*DEG*_*(s)*: Linearized first-order transfer function of DEG
*ΔP*_*DEG*_: Changes of DEG output power
*C(s)*: Controller output
*T*_*G*_: Governor time constant
*T*_*T*_: Turbine time constant
*R*: Speed control coefficient
*μ*_*DEG*_: Limitations the power increase
*δ*_*DEG*_: Limitations the ramp rate
*M*: Inertia constant
*D*: Load damping coefficient (= *ΔPD*/*Δ**F*)
*ΔPD*: Urban load demand
*G*_*FC*_*(s)*: Linearized first-order transfer function of FC
*ΔP*_*FC*_: Changes of FC output power
*K*_*FC*_: FC gain
*T*_*FC*_: FC time constant
*T*_*IN*_: Inverter time constant
*T*_*IC*_: Interconnection device time constant
